# Dual-Network Aerogel-Based Thermal-Safety Management System Design for Electric-Aircraft Battery Packs: Efficient Heat Management and Runaway Protection

**DOI:** 10.1007/s40820-026-02296-4

**Published:** 2026-07-21

**Authors:** Jie Yang, Yueyue Xiao, Mingyuan Yan, Xu Huang, Longlong Li, Zhongxin Zhang, Xudong Cheng, Heping Zhang, Yuelei Pan

**Affiliations:** 1 State Key Laboratory of Fire Science, University of Science and Technology of China, Hefei, 230027 People’s Republic of China; 2 School of Public Security and Emergency Management, Anhui University of Science and Technology, Hefei, 231131 People’s Republic of China; 3 Institute of Advanced Technology, University of Science and Technology of China, Hefei, 230027 People’s Republic of China

**Keywords:** Dual-network aerogel, High-efficiency thermal management, Synergistic regulation of thermal insulation and heat dissipation, Superinsulating material, Thermal protection

## Abstract

Electric-aircraft lithium-ion batteries, characterized by high-rate charge/discharge and high energy density, impose stringent thermal-safety requirements in confined compartments. However, conventional thermal-management or insulation strategies generally struggle to simultaneously enable efficient heat dissipation during routine operation and thermal runaway propagation (TRP) suppression under abuse conditions. Herein, a carbon aerogel–silica–alumina aerogel sheet–carbon aerogel dual-network aerogel (CA&SAAS) is fabricated through in-situ deposition and integrated supercritical drying and is further coupled with a cold plate (CP) to construct a battery thermal-safety management system (BTSMS). CA&SAAS exhibits a bulk density of 0.275 gcm⁻^3^ and a specific surface area of 626.37 m^2^g^⁻1^. The silica–alumina aerogel sheet (SAAS) core retains thermal conductivity of 0.0652 Wm^⁻1^K^⁻1^ at 1000 °C, while CA&SAAS exhibits a room-temperature thermal conductivity of 0.019 Wm^⁻1^K^⁻1^. During four 1C cycles, BTSMS limits *T*_max_/*ΔT*_max_ to 34.9/1.8 °C, reducing peak temperature and temperature nonuniformity by 46.5% and 56.1%, respectively. A predictive model shows optimized CP parameters constrain *T*_max_ to around 45 °C at 3C. Thermal runaway tests conducted in the confined space of a full-scale aircraft cargo compartment demonstrate that the BTSMS effectively interrupts TRP in a confined three-cell module, providing a solution for the design of BTSMSs for electric-aircraft battery packs.

**Supplementary Information:**

The online version contains supplementary material available at 10.1007/s40820-026-02296-4.

## Highlights

- A sandwich dual-network aerogel (CA&SAAS) is fabricated via in situ deposition and integrated supercritical drying; the SAAS core retains a low thermal conductivity of 0.0652 Wm^⁻1^K^⁻1^ at 1000 °C.
- Battery thermal-safety management system (BTSMS) enables efficient thermal regulation, limiting *T*_max_/*ΔT*_max_ to 34.9/1.8 °C at 1C, while simulations predict *T*_max_ around 45 °C under optimized 3C operation.
- BTSMS effectively interrupts thermal runaway propagation in a confined three-cell aircraft-module test through CA/CP-assisted heat dissipation and SAAS-enabled intercell thermal blocking.

**Supplementary Information:**

The online version contains supplementary material available at 10.1007/s40820-026-02296-4.

## Graphical abstract

**Figure:**
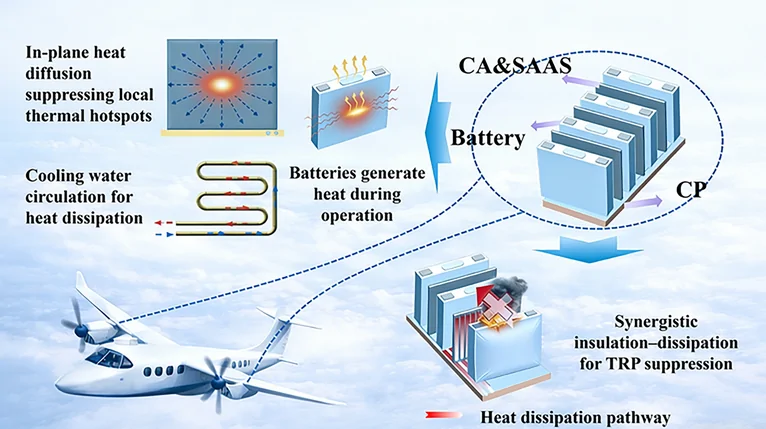

## Introduction

Amid the accelerating global shift toward low-carbon energy systems, electric aviation is emerging as a key route to sustainable transportation. Electrified flight platforms—particularly electric vertical takeoff and landing (eVTOL) aircraft—offer compelling advantages, including zero tailpipe emissions, low noise, and high maneuverability, and are now advancing rapidly toward commercial deployment [1, 2]. Lithium-ion batteries, featuring high specific energy, long cycle life, and excellent power capability, have become the core energy supply technology for electric propulsion systems [3, 4]. However, compared with ground applications, electric-aircraft batteries often experience sustained high-rate power demand during takeoff and landing, which makes rapid escalation of peak temperature more likely. Under adiabatic conditions, prior work reported a maximum temperature rise of 62 °C at 3C discharge, versus 36 °C at 1C, where C-rate denotes the charge/discharge current normalized to the nominal battery capacity (i.e., 1C corresponds to a current that fully charges or discharges the battery in 1 h) corresponding to a 26 °C increase in the peak temperature rise when moving from 1 to 3C [5]. Moreover, experiments interpreted using the Bernardi heat-generation framework indicate that the heat generation at 3C is 172% higher than that at 1C and 41.24% higher than that at 2C, with Joule heating being the dominant contribution [6]. These quantitative results underscore a pronounced rate-amplification effect on peak temperature under 3C-class high-power operation, thereby increasing the likelihood of localized overheating and subsequent thermal runaway (TR) [7]. Study has shown that during TR, the cell temperatures can rapidly exceed 900 °C [8]; TR can rapidly release intense heat and flammable gases and spread through battery modules within minutes, thereby triggering cascading thermal runaway propagation (TRP) events that threaten structural integrity and flight safety [9]. Therefore, developing advanced battery thermal-safety management systems that enable efficient heat dissipation during fast charge/discharge and actively suppress TRP under extreme scenarios is crucial for safe and reliable electric flight.

Existing battery thermal-management systems primarily rely on air [10–12], liquid cooling [13–15], phase-change materials (PCM) cooling [16–18], and heat-pipe cooling [19–21]. Among these, liquid cooling has been widely adopted because of its high heat-removal capability and excellent temperature controllability. The thermal performance of liquid cold plates can be effectively improved by optimizing channel configuration and operating parameters such as coolant flow velocity, channel geometry, and inlet temperature [22, 23]. Beyond the optimization of cooling structures, research on battery thermal behavior has gradually expanded from simple temperature-control experiments to system-level modeling and design-constraint analysis. Vinten et al. developed a full-vehicle transient thermal model for hybrid/electric vehicles, highlighting the importance of capturing highly transient battery thermal loads under practical driving conditions [24]. El-Sharkawy et al. further coupled a three-dimensional transient thermal framework with electrochemical and vehicle-dynamics models, enabling joint evaluation of battery thermal behavior, energy consumption, operating conditions, and degradation under modern drive cycles [25]. Arora et al. subsequently proposed a time–temperature analysis algorithm for estimating lithium-ion battery useful life, in which representative duty cycles, parking conditions, and high ambient temperatures were incorporated to assess life loss arising from both cyclic and calendar degradation [26]. In addition, El-Sharkawy et al. investigated the use of heat pipes for hybrid electric vehicle (HEV)/plug-in hybrid electric vehicle (PHEV) battery thermal management through a complete-vehicle transient thermal model, emphasizing that both the battery temperature level and temperature gradient must be controlled under coupled thermal loads from ambient air, airflow, exhaust radiation, and internal heat generation [27]. More recently, related engineering-oriented studies have further extended such system-level thinking toward the design of high-voltage battery systems and heat-shield structures for vehicle battery packs. The disclosed heat-shield design methodology, for example, explicitly links thermal-runaway analysis with ambient temperature, vehicle velocity, and design constraints, underscoring the growing trend toward integrated thermal-safety-oriented battery-pack design [28, 29].

Despite these advances, most existing studies still focus on the thermal regulation, durability prediction, or structural enhancement of conventional ground-vehicle battery systems. Their primary objective is to maintain acceptable temperatures during normal operation, whereas efficient heat dissipation under routine cycling and effective TRP suppression under extreme abuse are often treated separately. This separation becomes particularly problematic for electric-aircraft battery packs, because they operate in confined compartments where the heat-dissipation efficiency is intrinsically limited and heat accumulation is more severe during TR events. To address TRP hazards, current studies have mainly employed insulation materials such as asbestos layers [30], ceramic fibers [31], and aerogels [32]. Research had shown that using a 1-mm thick asbestos layer to separate adjacent batteries (Dimensions: 148 × 91.3 × 26.5 mm^3^, capacity: 27 Ah) can successfully prevent TRP in open spaces. However, asbestos poses severe health risks, including carcinogenicity, rendering it unsuitable for integration into electric-aircraft battery systems. Aerogels are nanoporous materials featuring low density, low thermal conductivity, high porosity, and high specific surface area and are thus widely used as thermal insulators [33]. A 2-mm aerogel sheet, for instance, can suppress TRP in 18650 cylindrical cells (diameter 18 mm, height 65 mm, 2.6 Ah) [32]. Despite the enhanced insulation, the inherently low thermal conductivity of high-performance aerogel layers can compromise heat dissipation during normal operation, potentially promoting heat accumulation and introducing latent thermal-safety risks [34]. Recent attempts to combine aerogel sheets with heat-dissipating components, such as graphene/reduced graphene oxide aerogels that construct thermally conductive networks followed by PCM impregnation to achieve both thermal energy storage and enhanced thermal conductivity [35, 36], have demonstrated improved thermal regulation and partial TRP mitigation. Nevertheless, such systems still face challenges associated with phase-change-induced instability, limited high-temperature durability, and insufficient suppression of TRP in confined spaces. Overall, purely insulating materials can delay TRP but inevitably compromise heat dissipation under routine operation. In contrast, thermally conductive or heat-storage composites improve heat redistribution to some extent, yet still suffer from limited stability in confined spaces and under high-temperature TR conditions.

To address these issues, this work proposes a battery thermal-safety management system (BTSMS) enabled by coupling a CP with a sandwich-structured carbon aerogel–silica–alumina aerogel sheet–carbon aerogel dual-network aerogel (CA&SAAS), which regulates battery temperature during routine cycling and suppresses TRP under extreme conditions. The design rationale and novelty of CA&SAAS are threefold: i) from the perspective of structural design, unlike PCM/graphene-aerogel hybrids that usually integrate conductive networks and phase-change components within the same composite phase through simple impregnation or physical combination, CA&SAAS adopts an integrated CA–SAAS–CA sandwich architecture fabricated via in-situ deposition and integrated supercritical drying. This one-piece aerogel structure preserves lightweight porous characteristics, which is more compatible with the mass-sensitive requirements of electric aircraft; ii) from the perspective of working mechanism, the intermediate Si/Al aerogel sheet exhibits low thermal conductivity and high-temperature resistance, enabling effective intercell thermal blocking, while the outer carbon aerogel skins provide enhanced in-plane heat spreading and facilitate heat dissipation in cooperation with the CP. In contrast, PCM-based systems mainly rely on latent-heat storage, and their heat-dissipation and thermal-barrier capabilities are limited under high-temperature TR conditions, making them less suitable for high-energy–density electric-aircraft battery packs; iii) from the perspective of application target, the proposed BTSMS is designed for confined electric-aircraft battery compartments, where high-rate heat dissipation during routine operation and TRP suppression under extreme abuse must be simultaneously achieved. Collectively, the dual-network strategy of CA&SAAS represents a structurally integrated and functionally partitioned design, alleviating the conventional conflict between thermal insulation and heat dissipation through CA-assisted heat spreading, CP-assisted heat extraction, and SAAS-enabled high-temperature thermal blocking. This provides a solution for improving the thermal-safety margins of electric-aircraft Li-ion battery packs under both routine and extreme conditions.

## Material and Methods

### Materials

Tetraethyl orthosilicate (TEOS), furfural, and resorcinol were purchased from Sinopharm Chemical Reagents Co., Ltd. Aluminum chloride hexahydrate (AlCl_3_·6H_2_O) was purchased from Shanghai Maclin Biochemical Technology Co., Ltd. Hexamethylenetetramine was purchased from Yunnan Jingrui Technology Co., Ltd. Ethanol (EtOH) and ammonia solution (NH_3_·H_2_O) were purchased from Chengdu Kelong Chemicals Co., Ltd. Mullite fibers were purchased from Shandong Luyang Thermal Insulation Materials Co., Ltd. Deionized water was used throughout the experiments.

### Preparation of CA&SAAS

First, TEOS, ethanol (EtOH), deionized water, and AlCl_3_·6H_2_O were mixed at a molar ratio of 160:1200:260:1 and magnetically stirred for 90 min to obtain a Si/Al hybrid sol. Subsequently, 0.5 molL⁻^1^ of NH_3_·H_2_O was added to adjust the pH. A mullite fiber felt was then impregnated with the as-prepared sol to form a composite sheet.

A resorcinol–furfural (RF) sol was subsequently prepared as the carbon-aerogel precursor via phenolic condensation coupled with sol–gel chemistry by mixing furfural (5 mL), anhydrous ethanol (10 mL), resorcinol (2 g), and hexamethylenetetramine (0.2 g) until fully dissolved. The resulting RF sol was in situ deposited onto both the top and bottom surfaces of composite sheet and aged in a sealed container at 70 °C to trigger interfacial polycondensation and gelation, thereby forming continuous organic gel “skin” layers while suppressing excessive infiltration into the composite sheet interior. After supercritical drying, the monolith preserved the integrated aerogel architecture; subsequent carbonization under an argon atmosphere converted the surface organic gel skins into carbon aerogel (CA), whereas the composite sheet interior transformed into a silica–alumina aerogel sheet (SAAS), yielding the sandwich-structured CA&SAAS composite.

### Characterization and Measurement

Scanning electron microscopy (SEM, HITACHI SU8010) was used to characterize the microstructure of CA&SAAS at multiple scales. The high-power X-ray diffractometer (XRD, TTR III) was employed to analyze the crystal structure of CA&SAAS, with scanning angles ranging from 10 to 80 °C and a scanning speed of 5 °Cmin^–1^. The X-ray photoelectron spectrometer (XPS, Thermo Scientific ESCALAB 250Xi) and the Nicolet 8700 Fourier transform infrared spectrometer (FTIR) were used to study the crystalline structure and chemical composition of CA&SAAS. The high-temperature resistance of CA&SAAS was verified using an infrared thermal camera (FLIR A655sc). The mechanical properties of CA&SAAS were measured according to GB/T 1964-1996 using a universal testing machine (Instron E3000K8953). The resilience properties of CA&SAAS was analyzed using a dynamic mechanical analyzer (Discovery DMA850). The thermal conductivity was measured using the plate heat flow meter method (DRPL-V, China, temperature range: 60–1000 °C). The chemical thermal stability was analyzed by using a thermogravimetric analyzer (TGA Q5000 IR) to heat from 25 to 800 °C at 10 °Cmin^−1^ under air flow. The Brunauer–Emmett–Teller (BET) specific surface area and pore structure of CA&SAAS were determined by N_2_ adsorption–desorption measurements using a surface area and porosity analyzer (Micromeritics ASAP 2460, USA).

### Experimental Arrangement

In this study, power lithium-ion cells supplied by Hubei Yiwei Power Co., Ltd. were used as the model system. The cells have a nominal capacity of 58 Ah with dimensions of 148.2 mm (L) × 95.2 mm (He) × 26.72 mm (W), and the key specifications are summarized in Table S1. All experiments were conducted under a full-scale aircraft environmental simulation chamber (photograph shown in Fig. S1). The main chamber features a flattened prismatic body with arcuate sidewalls, constructed from 8-mm stainless-steel plates. The inner dimensions are 467.0 cm in length, 112.0 cm in height, 122 cm in bottom width, and 300 cm in top width. Both the front and rear walls are equipped with pressure-resistant rectangular doors, each incorporating a rectangular tempered-glass viewing window. In addition, two rectangular tempered-glass viewing windows are installed on each curved sidewall to enable direct visual observation of the in-chamber experiments. A three-cell module was assembled in a confined chamber (Table S2). A liquid CP (Table S3) was installed at the bottom of the chamber and connected to a recirculating chiller (the relevant parameters of the recirculating chiller are listed in Table S4); water was used as the coolant with the set temperature maintained at 10 °C. A 2-mm-thick CA&SAAS sheet was inserted between adjacent cells. Three temperature points were arranged on each cell surface, and cycling tests were performed using a high-precision battery tester (NEWARE CTE-8008-5V200A). Specifically, all cells were charged using a 1C constant-current/constant-voltage (CC–CV) protocol and discharged under a constant-current (CC) mode. During charging, the cells were first charged at 58 A until the voltage reached 4.25 V, followed by constant-voltage charging at 4.25 V until the current decreased to 2.9 A. After a 30min rest, the cells were discharged at 58 A to the cut-off voltage of 2.8 V, followed by another 30min rest. This CC–CV charging protocol was used for all battery cycling and thermal runaway tests. The thermal-management performance test platform is shown in Fig. S1a.

To further assess the effectiveness of the BTSMS in suppressing TRP in confined spaces, TR was triggered by direct contact between a heating plate (Table S5) and the cell surface, following the procedure recommended in GB 38031-2020. Before the TR tests, all cells were charged to 100% state of charge (SOC) using a 1C constant-current/constant-voltage (CC–CV) protocol: the cells were first charged at 58 A–4.25 V and then maintained at 4.25 V until the current decreased to the cut-off current of 2.9 A. After charging, the cells were rested for 24 h to relax polarization, dissipate residual heat, and ensure consistent near-equilibrium initial conditions. The platform integrated multiple sensing modules: real-time surface temperatures during TR were recorded using a 7018 data acquisition module coupled with armored *K*-type thermocouples (Table S6), which were affixed to the cell surface using polyimide tape; voltage evolution was simultaneously monitored using a high-precision battery testing system (NEWARE CT-4008-5V6A); dynamic TRP behaviors were captured by a 4K ultra-high-definition video system (SONY FDR-AX700, 120 fps); and the three-dimensional temperature field inside the chamber was visualized using an infrared thermal imaging camera (FLIR A655sc). The TRP platform is illustrated in Fig. S1b, and the internal module layout and thermocouple positions are shown in Fig. S1c.

In this study, TR is defined as the condition where the cell surface temperature rise rate exceeds 1 °Cs⁻^1^ due to internal heat generation. This criterion is consistent with the thermal-runaway triggering condition specified in GB 38031-2025, where TR is identified when the monitored temperature rise rate satisfies dT/dt ≥ 1 °Cs⁻^1^ and persists for more than 3 s, in combination with either a voltage drop exceeding 25% of the initial voltage or the monitored temperature reaching the manufacturer-specified maximum operating temperature. If a higher temperature-rise-rate threshold were adopted, the identified TR onset could be delayed, potentially underestimating the response time required for early warning and thermal-safety intervention. Conversely, a lower threshold could lead to premature identification of TR, especially under externally driven heating conditions, thereby overestimating the severity of the early thermal response. If a fixed temperature threshold were used instead, the TR determination would become more sensitive to the initial cell temperature, ambient condition, cell chemistry, manufacturer-defined operating limits, and thermocouple location. More importantly, a fixed temperature threshold may not accurately capture the dynamic transition from externally induced heating to self-sustained exothermic reaction, which is the essential feature of TR. Therefore, the temperature-rise-rate criterion was adopted in this work to better identify the abrupt self-heating behavior associated with TR. Since all experimental groups were evaluated using the same criterion, the comparative conclusions regarding TR delay and TRP suppression remain robust.

### Experimental Uncertainty Analysis

The measurement uncertainty and experimental error in this work mainly originate from the accuracy of the testing instruments and remain within an acceptable range. The thermal conductivity of the samples at different temperatures was measured using a DRPL-V thermal conductivity analyzer, with a measurement error of less than ± 5%. During the charge–discharge cycling and thermal runaway tests, the cell surface temperature was recorded using *K*-type thermocouples, with a measurement error of ± 0.4%. The battery voltage was monitored by the battery testing system, with an error of ± 0.02%. The cold-plate temperature was controlled by a refrigerated circulator, with a temperature-control accuracy of ± 0.05 °C. Since all comparative groups were tested using the same measurement system, sensor arrangement, and data-acquisition method, these uncertainties do not alter the relative comparison among different configurations and do not affect the main conclusions regarding the thermal-management performance and thermal runaway propagation suppression capability.

## Results and Discussion

### Design Strategy of CA&SAAS

**Fig. 1:**
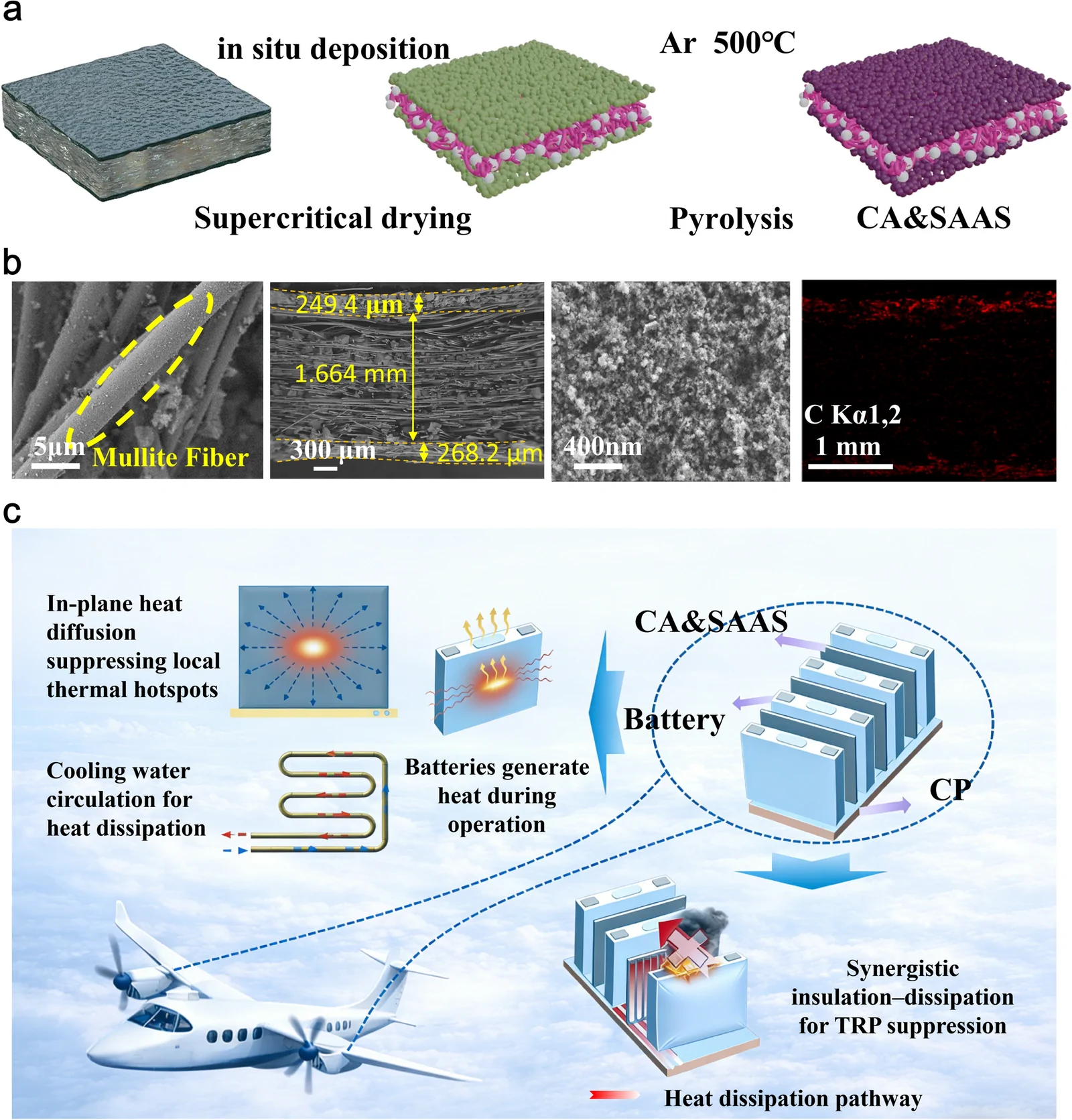
The Design strategy of CA&SAAS. **a** Synthesis process of CA&SAAS. **b** Micro-dual-network structure, cross-sectional SEM image with layer-thickness markings, and corresponding C elemental mapping of CA&SAAS. **c** Application concept of the BTSMS for confined compartments such as electric-aircraft battery packs

The fabrication of CA&SAAS can be summarized into three key stages, as illustrated in Fig. 1a. First, a mullite-fiber sheet is impregnated with a silica–alumina sol and aged to trigger in-situ gelation of the inorganic network within the fibrous scaffold, yielding a mechanically self-supporting silica–alumina composite sheet. Next, a resorcinol–furfural (RF) sol is deposited in-situ exclusively on the two outer surfaces of composite sheet; interfacial polycondensation and solidification generate continuous organic gel “skins” while effectively suppressing RF penetration into the interior silica–alumina gel network. The monolith is then shaped via an integrated one-step supercritical drying process, which maximally preserves hierarchical porosity and maintains interlayer interfacial integrity. Finally, thermal treatment under argon at 500 °C for 2 h converts the surface RF gel skins into carbon aerogel (CA) networks on both sides, whereas the central silica–alumina aerogel sheet (SAAS) remains as a refractory insulating framework, forming a “CA–SAAS–CA” (CA&SAAS) sandwich architecture; the photograph of the CA&SAAS is shown in Fig. S2. Here, the “dual-network” concept highlights the synergistic coupling between the two surface CA heat-spreading networks and the inner high-temperature SAAS insulation network.

The microstructural schematic, cross-sectional SEM images, and EDS elemental mapping of CA&SAAS are presented in Fig. 1b. The SEM images show that the Si/Al aerogel is uniformly coated on the surface of mullite fibers, forming a continuous and interconnected three-dimensional porous network together with the fiber skeleton. This structure not only preserves the nanoporous features of the aerogel, but also improves the structural stability of the material through the mullite-fiber framework. After the surface deposition–conversion process, a three-dimensional mesoporous framework assembled from cross-linked carbon nanoparticles is further formed on the SAAS surface, demonstrating that this strategy enables the construction of a continuous CA skin on SAAS. The outer CA layers are mainly formed through sol–gel chemistry, in which hexamethylenetetramine acts as an organic catalyst to regulate phenolic polymerization kinetics, enabling controlled nucleation of carbon nanoparticles and reinforcing the skin-like gel skeleton [37]. CA&SAAS exhibits a well-defined “CA–SAAS–CA” sandwich structure, in which the thicknesses of the two CA skins are 249.4 and 268.2 *μ*m, respectively, while the intermediate fiber-reinforced Si/Al aerogel layer has a thickness of 1.664 mm, giving a total thickness of 2.18 mm. The corresponding C elemental mapping further shows that the carbon signal is mainly enriched on the upper and lower surfaces of the material, whereas no obvious continuous carbon enrichment is observed in the central SAAS region. This indicates that the RF precursor is mainly confined to the SAAS surface during preparation and is subsequently converted into CA skins during carbonization, without obvious penetration into the internal Si/Al aerogel–mullite-fiber network. These results confirm the selective construction of the surface carbon layers and the structural integrity of the sandwich architecture in CA&SAAS. Figure 1c illustrates the application concept of the battery thermal-safety management system (BTSMS) for confined compartments such as electric-aircraft battery packs: under normal operation, in-plane heat spreading by the outer CA layers suppresses local thermal hotspots and improves temperature uniformity, while heat is continuously extracted through the CP and coolant circulation; upon TR, the central SAAS acts as a refractory barrier to impede lateral heat flux toward adjacent cells, and the CA–CP coupling provides a preferential heat-removal pathway, enabling a synergistic insulation–dissipation strategy that retards and suppresses TRP, thereby mitigating fire risk.

**Fig. 2:**
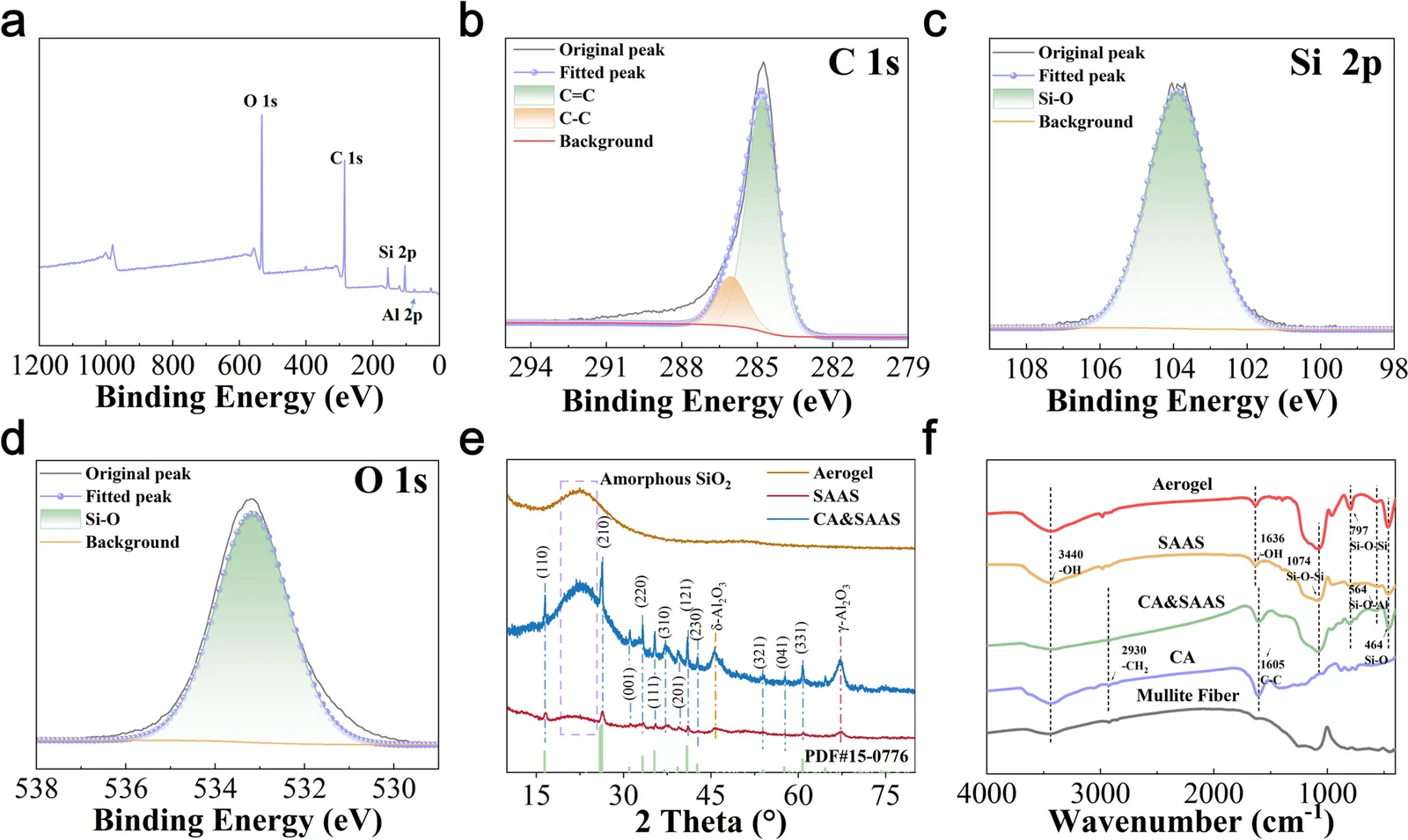
Characterization of the samples. **a** XPS survey of CA&SAAS. **b** C 1*s*, **c** Si 2*p*, **d** O 1*s* spectrum. **e** The XRD pattern of aerogel, SAAS and CA&SAAS. **f** FTIR spectrum of aerogel, SAAS, CA&SAAS, CA and mullite fiber

To clarify the chemical composition of CA&SAAS, X-ray photoelectron spectroscopy (XPS) was employed for characterization. As shown in Fig. 2a, the full-spectrum analysis detects characteristic peaks at O 1*s*, C 1*s*, Si 2*p*, and Al 2*p*, confirming the presence of C, O, and Si in CA&SAAS. The C 1*s* spectrum (Fig. 2b) reveals two characteristic peaks: 284.8 eV corresponding to *sp*^2^-hybridized C=C and 285.4 eV corresponding to *sp*^3^-hybridized C–C [32]. The characteristic peak at 103.6 eV for Si 2*p* spectrum confirms the chemical state of Si–O (Fig. 2c) [38]. Moreover, the O 1*s* spectrum (Fig. 2d) reveals a characteristic peak at 533.2 eV corresponding to the contributions of Si–O–Si [32], the Si 2*p* chemical state remains essentially unchanged, with no resolvable emergence of new silicon species or silicon-related crystalline signatures, while the O 1 *s* signal is predominantly attributable to bridging/structural oxygen within the aluminosilicate network. Collectively, these results indicate that CA deposition preserves the local chemical environment of the Si–Al–O skeleton and enables a stable surface-level coexistence of the carbon coating and the inorganic aluminosilicate framework. The XPS spectra of CA&SAAS closely mirror those of the aerogel (Fig. S3) and SAAS (Fig. S4). Both the survey and high-resolution scans consistently display the characteristic aluminosilicate signals (Si 2*p*, Al 2*p*, and O 1*s*) with essentially unchanged peak positions and line shapes, indicating that CA deposition preserves the chemical state of the underlying Si–Al–O network. The primary difference is the additional carbon contribution from the CA coating, giving rise to a surface-level spectral signature that reflects the coexistence of the carbon layer and the aluminosilicate framework. XPS analysis further verifies the presence of CA and Si/Al aerogel, confirming the formation of the dual-network structure.

The crystal structure of aerogel, SAAS, and CA&SAAS was characterized by XRD. As shown in Fig. 2e, aerogel exhibits broadened characteristic peaks around 22°, which can be attributed to the amorphous silica [39]; both SAAS and CA&SAAS retain the amorphous SiO_2_, while a set of reflections emerges at 16.5°, 26.2°, and 33.2° (together with weaker peaks across 30°–70°), which match well with the mullite reference (PDF#15-0776) [40]. The characteristic diffraction peak appearing near 23° in CA&SAAS corresponds to the (002) crystal plane of the amorphous carbon layer structure [41]. Due to the introduction of AlCl_3_·6H_2_O, trace amounts of *γ*-Al_2_O_3_ and *δ*-Al_2_O_3_ phases are detected in the samples, corresponding to characteristic diffraction peaks at 67.3° and 45.6°, respectively. It is worth noting that the intensity of the characteristic peaks of *γ*-Al_2_O_3_ and *δ*-Al_2_O_3_ in CA&SAAS significantly increases, indicating that the high-temperature pyrolysis process effectively promotes the crystallization transformation of Al_2_O_3_ [42].

FTIR was used to systematically characterize the structural features of mullite fibers, Si/Al aerogel, SAAS, CA, and CA&SAAS composites. As shown in Fig. 2f, all samples exhibit significant characteristic peaks at 3440 cm⁻^1^, and the characteristic peaks at 1630 cm⁻^1^ for Si/Al aerogel and SAAS can be attributed to the stretching and bending vibrations of –OH, which confirms the hydrophilic nature of the materials. Notably, the characteristic absorption peaks at 2930 cm⁻^1^ for CA and CA&SAAS correspond to the stretching vibrations of C–H, indicating that these peaks are related to residual organic precursors during the preparation of CA and CA&SAAS. Moreover, the characteristic peaks observed at 1605 cm⁻^1^ for both CA and CA&SAAS can be attributed to the bending vibrations of C=C, suggesting the aromatic ring structure of CA [43]. In the low-frequency region, Si/Al aerogel, SAAS, and CA&SAAS exhibit typical asymmetric Si–O–Si stretching vibration characteristic peaks at 1079, 797, and 464 cm⁻^1^, respectively. The Si–O–Al characteristic peak appearing at 564 cm⁻^1^ indicates that AlCl_3_·6H_2_O has successfully transformed into a stable Si–O–Al chemical structure in Si/Al aerogel, SAAS and CA&SAAS [44].

### Mechanical Properties of CA&SAAS

**Fig. 3:**
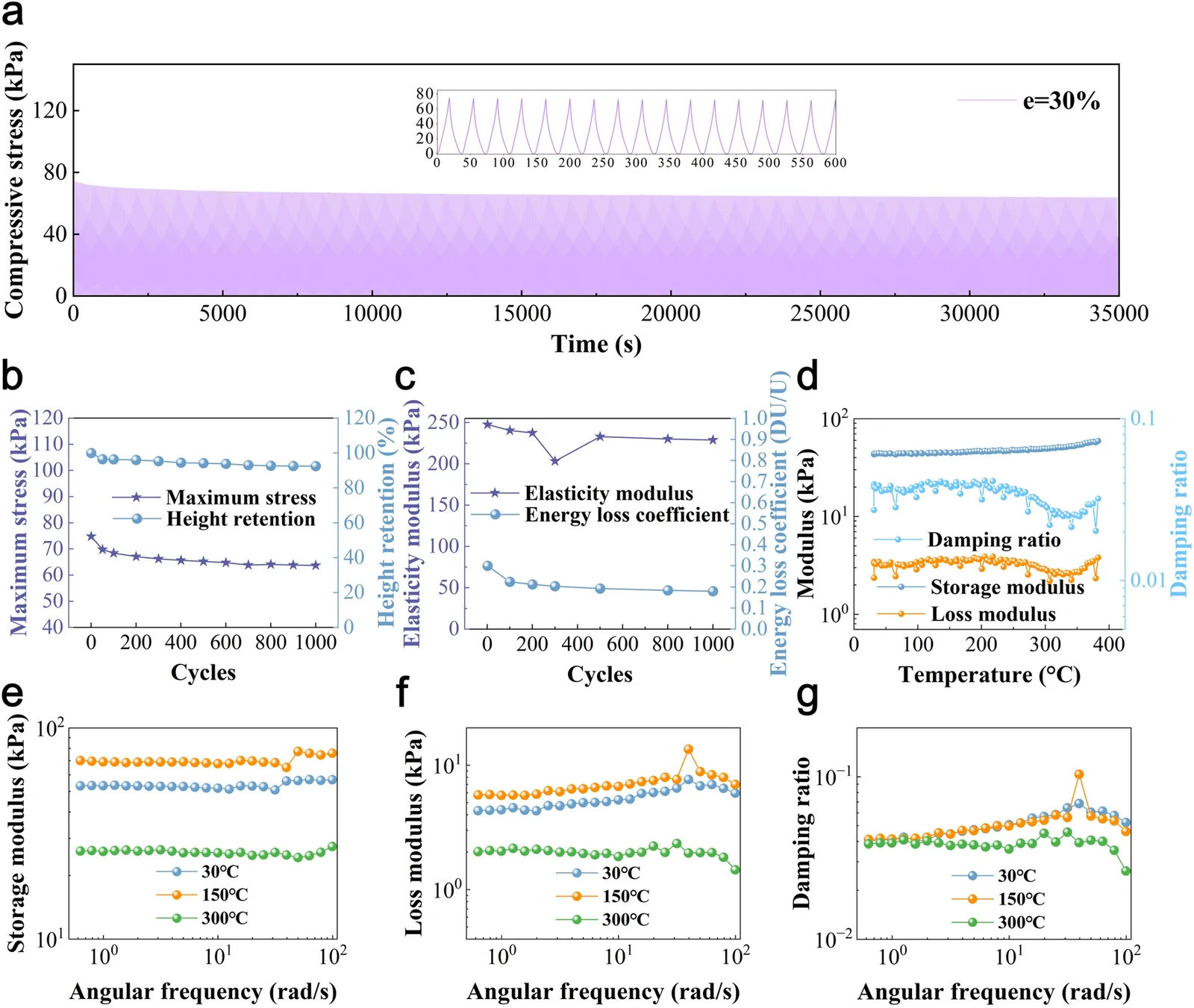
Mechanical properties of CA&SAAS. **a** Stress–time curves of the CA&SAAS in 1000 loading–unloading fatigue cycles. **b** Maximum stress and height retention. **c** Elastic modulus and energy loss coefficient. **d** Temperature dependence of the storage modulus, loss modulus, and damping ratio from 30 to 400 °C. **e ~ g** Storage modulus, loss modulus, and damping ratio of CA&SAAS at 30 °C, 150 °C, and 300 °C under angular frequencies ranging from 0.1 to 100 rad s⁻^1^

During flight, electric aircraft are continuously subjected to frequent, high-frequency vibration loads. As a key shock-absorbing spacer and thermal-protection component in lithium battery packs, the load-bearing capability and fatigue-resistant stability of aerogels directly affect cell service life and operational safety. Therefore, the universal testing machine (Instron E3000K8953) was used to perform 1000 loading–unloading cycles of compression tests on CA&SAAS at a high compression rate of 30%. As shown in Fig. 3a, CA&SAAS exhibits effective plastic deformation throughout the compression test, demonstrating its structural stability. Furthermore, parameters such as high retention rate, maximum stress, elastic modulus, and energy loss coefficient over the 1000-cycle process were analyzed. CA&SAAS maintained the maximum stress of 63.75 kPa and a height retention rate of 92.5% (Fig. 3b), confirming its outstanding compressive strength and elastic resilience. Based on the stress–strain curves, the elastic modulus and energy loss coefficient of CA&SAAS were calculated (Fig. 3c). After 300 cycles, the elastic modulus reaches 203.26 kPa, while the energy loss coefficient is 0.203. Both values gradually stabilize, indicating favorable elasticity and fatigue resistance.

To further elucidate the viscoelastic behavior of CA&SAAS, DMA was performed to quantify the storage modulus (G′), loss modulus (G″), and damping metric (defined in structural dynamics as ζ = tan*δ*/2 = G″/(2G′)). Under a constant frequency, the temperature-dependent response was examined from 30 to 400 °C (Fig. 3d). G′ remains within 40–60 kPa and G″ stays at 2–3 kPa throughout the entire temperature window, with no abrupt transitions or discernible relaxation peaks, indicating a stable mechanical response and structural integrity over a wide temperature range. Isothermal frequency sweeps were then conducted at 30, 150, and 300 °C over 0.1–100 rad s⁻^1^ (Fig. 3e–g). At 30 °C, G′ is 51–53 kPa and G″ is 4–6 kPa; at 150 °C, G′ is 67–70 kPa and G″ is 5–7 kPa; at 300 °C, G′ is 25–26 kPa and G″ is around 2 kPa. Notably, both G′ and G″ exhibit only minor variations with frequency at each temperature, displaying a plateau-like behavior and thus a weak frequency dependence. Meanwhile, ζ remains below 0.057 across the entire frequency domain, suggesting predominantly elastic deformation with low mechanical dissipation. This behavior is attributed to the 3D network jointly constructed by the Si/Al aerogel and mullite fibers: the continuous fibrous skeleton provides stable load-bearing pathways, while aerogel bridging and interfacial constraints promote stress delocalization and suppress local stress concentrations, thereby enhancing shape recoverability and structural resilience under cyclic loading—critical for long-term operation in the high-frequency vibration environment of electric aircraft.

### Thermal Stability and Insulation Performance of CA&SAAS

**Fig. 4:**
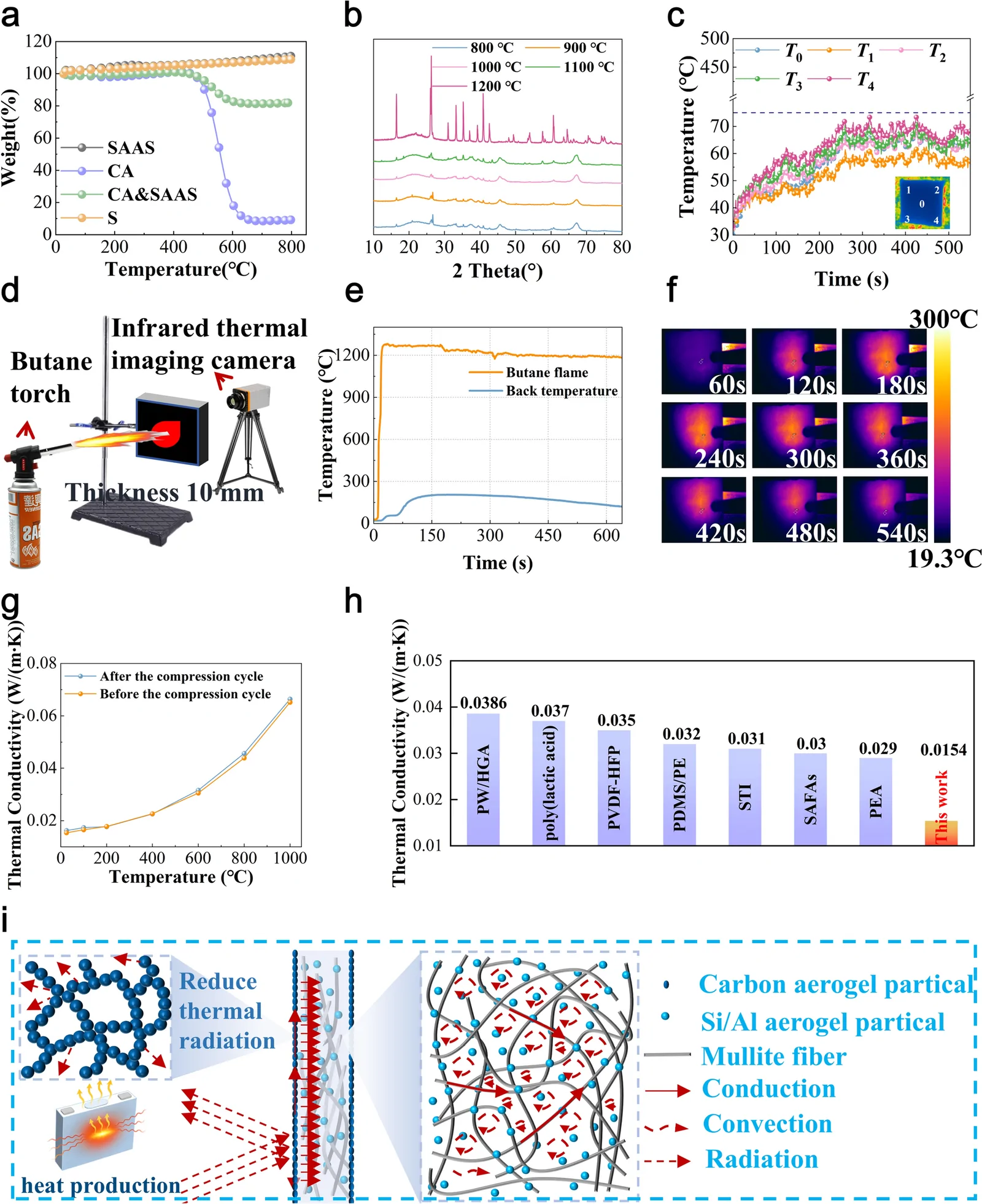
High-temperature resistance of SAS. **a** TG. **b** XRD patterns after heat treatment at 800 -1200 °C for 2 h. **c** Temperature rise at different points during hot plate processing. **d** Schematic diagram of backside temperature rise captured by infrared thermography during butane torch jet process. **e** Backside temperature rise curve. **f** Backside view of CA&SAAS during heating. ** g** Thermal conductivity of SAAS before and after compression cycles from 25 to 1000 °C. **h** Thermal conductivity of SAAS at room temperature compared with other previously reported heat-dissipating aerogel insulation sheets under ambient conditions (25 °C). **i** Thermal insulation mechanism diagram of CA&SAAS

To evaluate the thermochemical stability of CA&SAAS in air, thermogravimetric analysis (TGA) was performed on mullite fiber sheets, SAAS, CA, and CA&SAAS (Fig. 4a). Within the temperature range of 25–800 °C under an air atmosphere, mullite fiber and SAAS exhibited a slight mass increase, which can be attributed to the formation of Al_2_O_3_ and SiO_2_ from residual trace elements (Al and Si) in mullite fiber sheets under high-temperature oxidation conditions. Notably, both CA and CA&SAAS showed relatively stable mass change curves from 25 to 450 °C, confirming the excellent thermochemical stability of CA&SAAS. As the temperature increases to 450–800 °C, thermal decomposition of –CH_2_– and C–C in CA occurs, resulting in periodic mass loss in CA&SAAS. However, the observed mass loss rate in this temperature range remained very low, further demonstrating the high-temperature structural stability of CA&SAAS at the molecular level.

To systematically evaluate the high-temperature tolerance and structural stability of CA&SAAS under thermal exposures relevant to battery packs in electric, X-ray diffraction (XRD) analysis was conducted on CA&SAAS subjected to isothermal treatment at 800, 900, 1000, 1100, and 1200 °C for 2 h. As shown in Fig. 4b, the diffraction peaks corresponding to the *γ*-Al_2_O_3_ phase begin to intensify at 900 °C and exhibit a more pronounced increase as the temperature rises to 1000–1100 °C. Notably, in the XRD pattern of CA&SAAS treated at 1100 °C, a sharp diffraction peak appeared at 57.52°, corresponding to the (116) crystal plane of *α*-Al_2_O_3_, which matches the PDF#46-1212 standard card [45], indicating the occurrence of a *γ* → *α* phase transition. After heat treatment at 1200 °C, the relative content of mullite fibers increased significantly, and most *γ*-Al_2_O_3_ was converted into the thermodynamically stable *α*-Al_2_O_3_ phase [42]. It was also worth noting that the SiO_2_ component retained its amorphous nature across the entire temperature range, confirming the excellent chemical and thermal stability of the material.

To further evaluate the structural stability of SAAS after high-temperature treatment, the density variation after thermal treatment at different temperatures for 2 h was measured (Fig. S5). The densities of SAAS after treatment at 25, 200, 400, 600, 800, and 1000 °C were 0.0265, 0.0288, 0.0281, 0.0273, 0.0270, and 0.0287 gcm⁻^3^, respectively. Overall, the density of SAAS fluctuated only slightly within the range of 0.0265–0.0288 gcm^-^^3^, with a maximum variation of 8.68%. This result indicates that SAAS does not undergo obvious shrinkage, collapse, or densification after high-temperature treatment, confirming that the mullite-fiber-reinforced Si/Al aerogel network maintains good structural integrity under high-temperature conditions.

In addition, the overall density of CA&SAAS was 0.275 g·cm^-3^, indicating that the composite retains a lightweight porous characteristic after the introduction of the CA skins, while gaining the structural reinforcement and heat-spreading function provided by the surface carbon aerogel layers. The pore-structure parameters of CA&SAAS were further quantified by N_2_ adsorption–desorption analysis. The BET-specific surface area and total pore volume of CA&SAAS were 626.37 m^2^g^-1^ and 2.56 cm^3^g^-1^ (Fig. S6), respectively. The BJH adsorption and desorption cumulative pore volumes were 2.55 and 2.55 cm^3^g^-^^1^, respectively, while the average pore diameter was mainly in the range of 13–16 nm (Fig. S7). These results confirm that CA&SAAS possesses a high-surface-area, large-pore-volume, mesopore-dominated porous structure. The dominant mesopore distribution within 2–50 nm provides a structural basis for suppressing gas-phase thermal conduction through the Knudsen effect, because nanoscale pores enhance gas–solid wall collisions and restrict gas molecule transport. Based on this stable and highly porous structure, the thermal conductivities of SAAS and CA&SAAS were further systematically measured to quantitatively evaluate their ability to suppress heat transfer between adjacent modules as thermal barriers.

On this stable and highly porous structural basis, infrared thermography was employed to dynamically track the macroscopic thermal-response behavior of CA&SAAS under external thermal stimuli. Firstly, CA&SAAS was placed on a 200 °C heating plate, and the temperature evolution at different surface points was monitored. After approximately 9 min, the surface temperature reached its peak value. In the steady state, the average surface temperature remained below 70 °C, resulting in a significant temperature difference of 130 °C (Fig. 4c), confirming the excellent high-temperature resistance of CA&SAAS. Furthermore, a continuous 11-min flame exposure test was conducted using a butane torch (flame temperature: 1200 - 1300 °C). Infrared thermal imaging data (Fig. 4d–f) revealed the temperature evolution on the back side of the material during the thermal shock and subsequent heat dissipation process. In the initial stage, a steep temperature gradient was rapidly established, with the back-side temperature peaking at 21 °C. Subsequently, heat exchange occurred between CA and the surrounding environment, and the temperature gradually decreased to a steady-state value of 118 °C. These experimental results quantitatively demonstrated CA&SAAS's thermal response behavior under thermal conditions, providing solid evidence for understanding the high-temperature resistance mechanism of CA&SAAS.

To quantitatively characterize its thermal insulation performance, the thermal conductivity of SAAS was systematically measured across different temperature ranges to quantitatively evaluate its ability to suppress heat transfer between adjacent modules as a thermal barrier. As shown in Fig. 4g, SAAS exhibits an ultralow thermal conductivity of 0.0154 Wm^-1^K^-1^ at room temperature and retains a low value of 0.0652 Wm^-1^K^-1^ even when the temperature increases to 1000 °C, demonstrating excellent high-temperature insulation performance. In addition, the through-thickness thermal conductivity of CA&SAAS was measured to be 0.019 Wm^⁻1^K^⁻1^ at room temperature, indicating that the composite still maintains low transverse thermal conductivity after the introduction of the CA skins. This result confirms that the CA layers provide surface heat-spreading capability without substantially compromising the thermal-barrier function of the SAAS core. Considering that battery-pack insulation materials are inevitably subjected to sustained mechanical stresses during aircraft takeoff, landing, taxiing, and flight, it is essential to further evaluate their insulation stability after compression. Therefore, to simulate the mechanical compressive loads encountered during service, SAAS was subjected to 1000 compression cycles at 30% strain, after which its thermal conductivity before and after compression was measured over 25–1000 °C. The results show that, after 1000 compression cycles, the room-temperature thermal conductivity of SAAS increased only slightly from 0.01539 to 0.01622 Wm^⁻1^K^⁻1^. At 100 °C, the thermal conductivity increased marginally from 0.0165 to 0.0174 Wm^⁻1^K^⁻1^, whereas at 1000 °C, no obvious difference was observed before and after compression. Moreover, the thermal-conductivity curves before and after compression show highly consistent temperature-dependent trends across the entire measured range. These results indicate that SAAS maintains stable thermal-insulation performance after repeated mechanical compression, further confirming its excellent mechanical stability and high-temperature thermal-protection reliability. It should be noted that the thermal conductivity reported here represents the effective through-thickness thermal conductivity of the SAAS composite rather than the intrinsic thermal conductivity of the pure Si/Al aerogel phase. Although mullite fibers possess a relatively high intrinsic thermal conductivity, they are distributed as a loose and interlaced fiber network in SAAS and do not form continuous straight heat-conduction pathways. Meanwhile, the Si/Al co-precursor aerogel network improves high-temperature structural stability by forming stable Si–O–Al linkages and suppressing thermally induced sintering/shrinkage of the silica skeleton, thereby helping preserve the porous architecture at elevated temperatures [32]. This structural stability, together with the high porosity and mullite-fiber-reinforced tortuous heat-transfer pathways, contributes to the low effective thermal conductivity of SAAS at 1000 °C.

The thermal conductivity of SAAS at room temperature was compared with the room-temperature thermal conductivities of other aerogel-based thermal insulation sheets with heat dissipation capabilities reported in previous studies. As shown in Fig. 4h, SAAS exhibited a lower thermal conductivity than other aerogel materials reported before, such as the composite thermal insulation (STI) board (copper//silica dioxide aerogel//copper) [46], paraffin wax/hybrid graphene aerogel (PW/HGA) [47], polyethylene aerogel (PEA) [48], silica-alumina nanofibrous aerogels (SAFAs) [49], poly(dimethylsiloxane) with polyethylene (PDMS/PE) [50], poly(vinylidene fluoride-hexafluoropropylene) (PVDF-HFP) [51], and poly(lactic acid) [52]. This further confirmed the strong application potential of SAAS in suppressing TRP.

To further clarify the thermal-insulation mechanism of CA&SAAS, we establish a heat-transfer model consistent with its hierarchical multilayer porous architecture to capture the heat-transport and dissipation pathways within the composite, thereby providing a theoretical basis for quantitatively interpreting its insulation performance. CA&SAAS is treated as a multilayer slab comprising two outer CA skins and an intermediate SAAS. Because the in-plane dimensions are much larger than the total thickness, the dominant heat flow in the insulation tests can be approximated as one-dimensional along the thickness direction a (0 ≤ *a* ≤ *d*), where *d* = *d*_CA,1_ + *d*_SAAS_ + *d*_CA,2_.

For each layer m (m represents any layer in CA&SAAS, such as CA or SAAS), energy conservation combined with Fourier’s law gives:

1 $$\rho_{m} c_{m} \frac{{\partial T_{m} }}{\partial t} = \frac{\partial }{\partial a}\left[ {\lambda_{{m,{\mathrm{eff}}}} \left( T \right)\frac{{\partial T_{m} }}{\partial a}} \right]$$

where ${\rho}_{m}$, ${c}_{m},$ and ${\lambda}_{m,eff}\left(T\right)$ denote the density, specific heat, and effective thermal conductivity, respectively [53].

At an internal interface *a* = *a*_int_:

2 $$T_{m} \left( {a_{{\mathrm{int}}}^{ - } ,t} \right) = T_{n} \left( {a_{{\mathrm{int}}}^{ + } ,t} \right)$$

3 $$- \lambda_{{m,{\mathrm{eff}}}} \left( T \right)\frac{{\partial T_{m} }}{\partial a}\left( {a_{{\mathrm{int}}}^{ - } } \right) = - \lambda_{{n,{\mathrm{ef}}f}} \left( T \right)\frac{{\partial T_{n} }}{\partial a}\left( {a_{{\mathrm{int}}}^{ + } } \right)$$

where a_int_ represents the interface position, and +/- indicates the right/left side of the interface, ${q}^{{\prime}{\prime}}=-\lambda \frac{\partial T}{\partial a}$ [53].

For highly porous aerogel-type media, pore-scale convection is strongly suppressed, ignoring any possible cross-coupling effects between them; thus, ${\lambda}_{m,eff}$ can be expressed as [54]:

4 $$\lambda_{{m,{\mathrm{eff}}}} = \lambda_{{{\mathrm{solid}},m}} + \lambda_{{{\mathrm{gas}},m}} + \lambda_{{{\mathrm{rad}},m}}$$

When the characteristic pore size *D* becomes comparable to or smaller than the molecular mean free path l $\left( {{\mathrm{Kn}} = \frac{l}{D} \ge 1} \right)$, gas–wall collisions dominate and the gaseous conductivity decreases markedly. A commonly adopted Knudsen-corrected form is:

5 $$\lambda_{{{\mathrm{gas}}}} = \lambda_{{{\mathrm{gas}},0}} \frac{\Phi }{1 + 2\beta Kn}$$

where ${\lambda}_{gas,0}$ is the free-gas thermal conductivity (excluding convection), Φ is the open porosity, and β is related to the energy accommodation at the gas–solid interface [38]. Because l depends on temperature and pressure (from kinetic theory), mesopore-dominated CA&SAAS (2–50 nm) at ambient pressure yields large *Kn*, thereby strongly suppressing ${\lambda}_{gas}$, consistent with the pore-size distribution in Fig. S7.

At elevated temperatures, radiative transfer within porous media can be treated via the Rosseland diffusion approximation:

6 $$q^{\prime\prime}_{{{\mathrm{rad}}}} = - \lambda_{{{\mathrm{rad}}}} \left( T \right)\frac{dT}{{da}}$$

7 $$\lambda_{{{\mathrm{rad}}}} \left( T \right) = \frac{{16n^{2} \sigma T^{3} }}{{3\beta_{R} }}$$

where *n* is the refractive index, *σ* is the Stefan–Boltzmann constant, and ${\beta}_{R}$ is the Rosseland mean extinction coefficient. A larger ${\beta}_{R}$ directly lowers ${\lambda}_{rad}\left(T\right)$, suppressing radiative heat transfer [55]. In addition, the CA skins possess strong infrared attenuation, which can be described by the Beer–Lambert law:

8 $$I\left( a \right) = I_{0} e^{ - \beta \prime a}$$

where $\beta {\prime}$ is the mean extinction coefficient. This mechanism accounts for the “first-stage radiation shielding” of CA during flame exposure, consistent with the established role of carbon/opacifiers in reducing radiative transport in aerogel composites [56].

The model clarifies that the superior high-temperature tolerance of CA&SAAS arises from the synergistic suppression of ${\lambda}_{gas}$ by nanoscale pores (Knudsen effect), suppression of radiative transfer ${\lambda}_{rad}$ via high-extinction CA skins, and the tortuous solid conduction pathway within the mullite-fiber–Si/Al aerogel network [57]. Figure 4i schematically links the proposed heat-transfer model with the microstructural origins of the insulation behavior of CA&SAAS. Under external heating, the outer CA skin acts as a radiative shield: Its high extinction capability attenuates incident thermal radiation at the surface, thereby suppressing the radiative term ${\lambda}_{rad}$ (Eqs. 6–8) and mitigating direct radiative penetration into the interior [58]. The remaining heat is then conducted into the intermediate SAAS layer, which consists of a 3D porous framework formed by mullite fibers decorated with Si/Al aerogel particles. Consistent with the pore-size distribution in Fig. S7, the SAAS-dominated mesopores (2–50 nm, with a main population centered at 15–25 nm and most pores below 50 nm) strongly inhibit pore-gas heat transfer via the Knudsen effect, rendering pore convection negligible and markedly reducing ${\lambda}_{gas}$ (Eq. 5) [59]. Meanwhile, the slender and tortuous mullite-fiber skeleton elongates the effective conduction pathways and limits the solid contribution ${\lambda}_{solid}$. Upon reaching the cold-side CA skin, the residual heat flux is further dissipated/attenuated, establishing a multi-stage, synergistic insulation process across the CA–SAAS–CA architecture, which is consistent with the pronounced through-thickness temperature drop observed experimentally [60].

### Thermal-Management Performance of BTSMS

In electric-aircraft platforms, lithium-ion battery packs are often integrated into space-constrained compartments with stringent thermal-management conditions; therefore, heat accumulation generated during charge–discharge can markedly raise cell temperatures and accelerate destabilizing reactions, posing a potential risk of TR [61, 62]. To address the reduction in heat dissipation efficiency caused by the incorporation of aerogel insulation materials. To prevent heat accumulation within the lithium-ion battery pack, a BTSMS by coupling CA&SAAS with a CP is designed. Through the contact between the CA and the CP, heat is rapidly dissipated from the battery pack. The BTSMS enables gradient regulation of the internal thermal field within the battery module. A bottom-mounted circulating liquid cooling configuration is employed, in which a serpentine-flow CP is arranged at the base of the battery pack. This setup enhances convective heat transfer and effectively increases the heat flux density, thereby improving overall thermal-management performance.

**Fig. 5:**
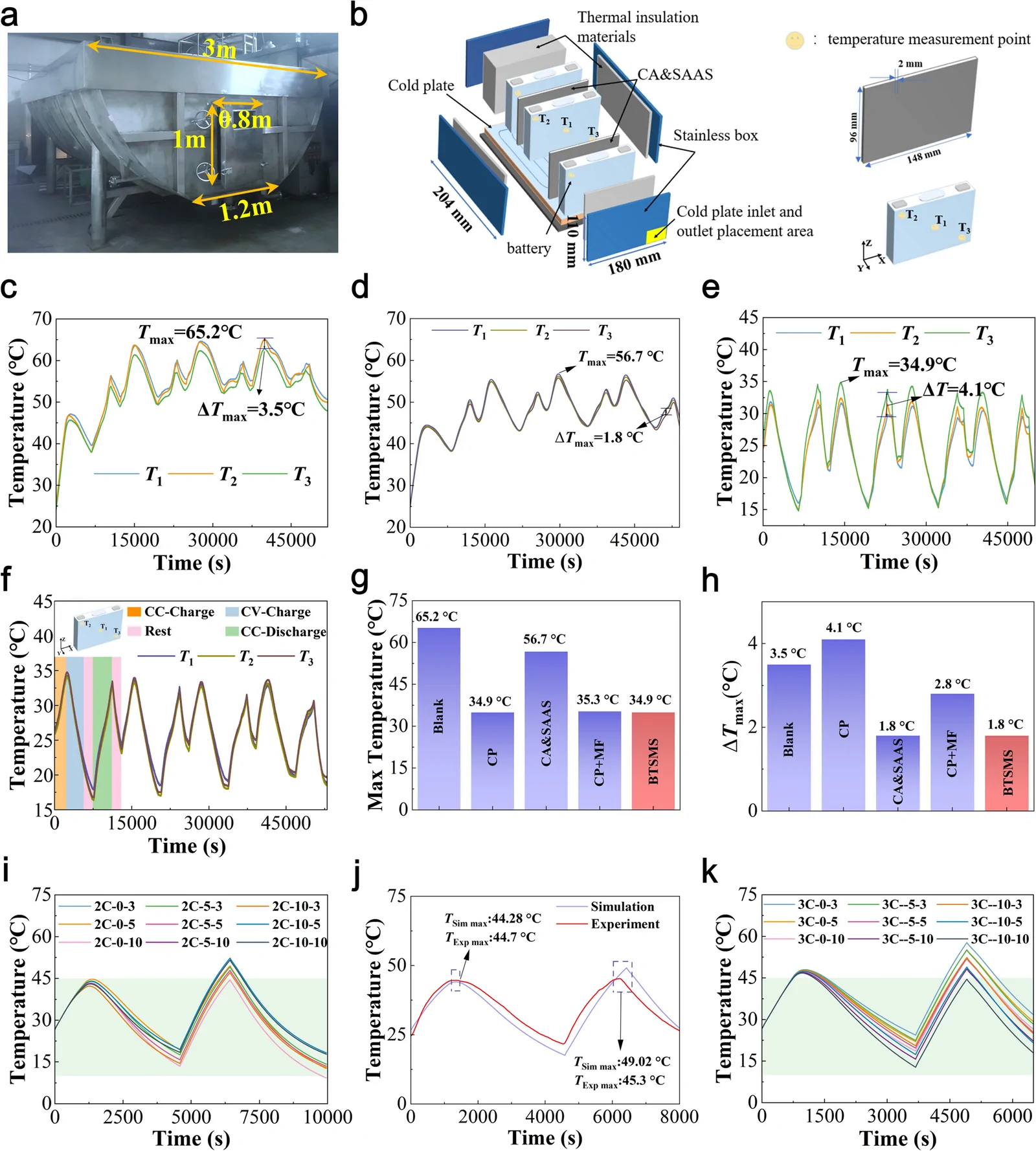
Thermal-management performance of CA&SAAS. **a** Actual images and dimensions of a full-scale aircraft environmental simulation chamber. **b** Battery pack layout diagram. The TR temperature evolution curves of **c** Blank, **d** CA&SAAS, **e** CP and **f** BTSMS. **g** Max temperature. **h** Max temperature difference. **i** Temperature rise curve at 2C rate. **j** Comparison chart of experimental and simulated data under 2C rate charge–discharge conditions. **k** Temperature rise curve at 3C rate

All experiments were conducted at ambient conditions (25 °C, 101 kPa) in a full-scale aircraft environmental simulation chamber (photograph shown in Fig. 5a). The actual layout inside the battery pack is shown in Fig. 5b. To systematically evaluate the thermal-management performance of different configurations and distinguish the respective contributions of CP and CA&SAAS in the BTSMS, five comparative groups were designed: Blank (without CA&SAAS or CP), CP (CP only), CA&SAAS (CA&SAAS only, without CP), CP+Mullite Fiber (CP+MF), and BTSMS. The Blank group was used to characterize the natural temperature rise of the battery module in a confined space without any thermal-management structure. The CP group was designed to evaluate the role of the liquid cold plate in heat-removal and peak-temperature reduction, while the CA&SAAS group was used to assess the passive thermal-regulation capability of the composite aerogel itself. In the CP+MF group, an equivalent-thickness needle-punched mullite-fiber felt was introduced to exclude the influence of intercell spacing and contact thermal resistance on the temperature response. The MF used here has a density of 0.028 g cm^⁻3^ and a thermal conductivity of 0.044 Wm^⁻1^K^⁻1^. The BTSMS group was used to validate the integrated thermal-management performance achieved by coupling CP with CA&SAAS.

During 1C charge–discharge cycling (the current–voltage curve is shown in Fig. S8), the Blank group (Fig. 5c) reaches a maximum temperature of 65.2 °C and a maximum temperature difference of 3.5 °C, indicating severe heat accumulation in the confined module without thermal management. After introducing CP (Fig. 5e), the peak temperature is markedly reduced to 34.9 °C, confirming that CP serves as the dominant heat-removal pathway for the module. However, because the cold plate is positioned at the cell bottom and induces localized strong cooling, the maximum temperature difference increases to 4.1 °C, indicating that liquid cooling alone may aggravate temperature-field nonuniformity while reducing the absolute peak temperature. In contrast, when CA&SAAS is used alone (Fig. 5d), the peak temperature remains 56.7 °C, suggesting that CA&SAAS cannot efficiently remove continuously generated heat in the absence of an active cooling pathway. Nevertheless, its maximum temperature difference is only 1.8 °C, demonstrating that the outer CA layers promote in-plane heat spreading and lateral heat redistribution, thereby improving surface-temperature uniformity.

When CP and CA&SAAS are coupled to form the BTSMS, the peak temperature is limited to 34.9 °C, while the maximum temperature difference remains as low as 1.8 °C (Fig. 5f), indicating that the BTSMS integrates the active heat-removal capability of CP with the in-plane temperature-homogenization capability of CA&SAAS. Moreover, the CP+MF group shows a peak temperature of 35.3 °C, close to that of the BTSMS, confirming that the altered intercell spacing and contact thermal resistance introduced by an equivalent-thickness spacer can affect the peak-temperature response. However, the maximum temperature difference of the CP+MF group remains 2.8 °C, notably higher than that of the BTSMS (Fig. S9). This is because the needle-punched mullite-fiber felt mainly consists of randomly interlaced fibers with limited inter-fiber contact points and lacks a continuous in-plane heat-conduction network. Therefore, it acts primarily as a spacer and passive thermal barrier, but cannot rapidly spread local heat laterally like the CA skins. Compared with the CP+MF group, the BTSMS further reduces the maximum temperature difference by 35.7%, indicating that the improved temperature uniformity is not merely caused by the geometric spacing or contact thermal resistance introduced by the 2-mm interlayer, but mainly arises from the synergistic effect of in-plane heat spreading by the CA skins and thermal blocking by the SAAS core. In addition, the in-plane thermal conductivity of CA&SAAS reaches 0.20 Wm^⁻1^K^⁻1^, approximately five times that of SAAS (0.04 Wm^⁻1^K^⁻1^), confirming that the CA skins provide an effective pathway for rapid lateral heat spreading over the cell surface. (Comparison of rapid in-plane heat-spreading capability is provided in S1.2.) Overall, during four 1C cycles, the BTSMS achieves a 46.5% reduction in peak temperature relative to the Blank group and a 56.1% reduction in temperature nonuniformity relative to the CP group, demonstrating its capability for simultaneous heat dissipation and temperature homogenization in confined battery modules (Fig. 5g-h).

The geometric model of the battery pack equipped with the BTSMS was constructed using SolidWorks and further cleaned, simplified, and preprocessed in ANSYS SpaceClaim; the processed geometry was then imported into STAR-CCM+ for mesh generation and subsequent conjugate heat-transfer simulations under different charge/discharge rates and CP set temperatures. Quantitative assessment of thermal-safety performance requires an explicit heat-generation model. During operation, cell heat generation primarily arises from the combination of irreversible heat (e.g., ohmic and polarization losses) and reversible entropic heat. In battery thermal management and CFD-based thermal simulations, the Bernardi equation is widely adopted as a transient heat-source model [63]; it links the electrical power imbalance to internal energy change via energy conservation and decomposes the total heat generation into irreversible and reversible (entropic) contributions. A commonly used form is:

9 $$q = \frac{1}{V}\left[ {I\left( {E - U} \right) + {\mathrm{IT}}\frac{\partial E}{{\partial T}}} \right] = \frac{1}{V}\left[ {I^{2} \left( {R_{j} + R_{p} } \right) + {\mathrm{IT}}\frac{\partial E}{{\partial T}}} \right]$$

Here, *q* is the volumetric heat-generation rate (W·m^−3^) and *V* is the cell volume. *I* denotes the current (positive for discharge and negative for charge) [64, 65]; *E* and *U* are the open-circuit voltage/electromotive force (OCV) and the terminal operating voltage, respectively. *R*_j_ and *R*_p_ represent the equivalent ohmic and polarization resistances, and *T* is the temperature. The term ${I}^{2}\left({R}_{j}+{R}_{p}\right)$ corresponds to irreversible heat generation arising from ohmic and polarization losses, whereas $IT\frac{\partial E}{\partial T}$ accounts for the reversible entropic heat contribution.

In the conjugate heat-transfer simulations performed in STAR-CCM+, heat generation was described using the Bernardi model. Each cell was treated as an equivalent homogeneous unit, and the volumetric heat-generation rate was implemented as a uniformly distributed internal heat source. The cell heat conduction was then coupled with heat conduction in the CP solid and with the incompressible flow and energy equations of the coolant, enabling prediction of the spatiotemporal evolution of the pack temperature field under different C-rates and CP set temperatures.

The hybrid pulse power characterization (HPPC) method was employed to quantify the cell internal resistance, enabling accurate determination of the charge/discharge direct current resistance (DCR) over a wide temperature range. The DCR variations measured at 15–35 °C are summarized in Figs. S12 and S13. Specifically, Fig. S12 shows the dependence of DCR on state of charge (SOC) and ambient temperature. The DCR is highly temperature-sensitive: increasing the temperature from 15 to 35 °C leads to a pronounced overall decrease. With respect to SOC, the DCR is markedly higher at low SOC (0–10%), becomes relatively flat at intermediate SOC (20%–60%), and slightly rises again toward high SOC. Similar trends are observed for both charge and discharge, while the discharge DCR (Fig. S13) is comparatively higher at low SOC. Figure S14 compares the experimental and simulated cell temperature profiles at 1C. The two curves overlap closely throughout the charge–rest–discharge-rest process; the temperature rise curves from the experiment and simulation exhibit a high degree of consistency, demonstrating the high reliability of the simulation.

The input and output parameters of the constructed model are summarized in S1.3, and the grid-independence analysis is provided in S1.4 and Table S7. Building on the validated model, we further extended the simulations to combined conditions of varying C-rates, CP set temperatures, and coolant flow rates. To achieve a lower coolant inlet temperature and enhanced heat-removal capability, the coolant was switched from water to a 20% ethylene glycol aqueous solution when the CP set temperature was reduced to 0 °C and below. Under the 2C condition (Fig. 5i; CP temperature of 0 ~ 10 °C with varied flow rates), the peak cell temperature predominantly falls within 45 ~ 50 °C, while a lower plate temperature combined with a higher flow rate further suppresses the maximum temperature to around 45 °C or slightly below. In particular, at a CP temperature of 0 °C and a flow rate of 10 L·min⁻^1^, the cell temperature during charge/discharge can be fully maintained within the optimal operating window. To further validate the reliability of the simulation prediction, a 2C charge–discharge experiment was conducted at a CP temperature of 5 °C and a coolant flow rate of 3 Lmin^-1^. (The current curve of the experimental process is shown in Fig. S15.) During charging, the simulated and experimental maximum temperatures were 44.08 and 44.7 °C (Fig. 5j), respectively, corresponding to an error of 1.4%. During discharging, the simulated and experimental maximum temperatures were 49.02 and 45.3 °C, respectively, corresponding to an error of 8.2%. The peak-temperature deviations between the simulation and experiment are both below 10%, indicating that the model can reasonably predict the main thermal response of the battery module under high-rate operation. These results further confirm that decreasing the CP temperature and increasing the coolant flow rate synergistically strengthen the heat-dissipation performance and thermal-regulation capability of the BTSMS.

Notably, under the more demanding 3C high-rate scenario (Fig. 5k), lowering the CP temperature to − 5 ~ − 10 °C markedly suppresses the peak module temperature, confining it to around 45 ~ 60 °C. When the CP temperature is further reduced to −10 °C and the coolant flow rate is increased to 10 L·min⁻^1^, the peak temperature during charge/discharge can be stably maintained at around 45 °C. Overall, these multi-condition simulations provide quantitative guidance for selecting key thermal-management parameters for electric-aircraft batteries (CP temperature, coolant flow rate, and operating C-rate), while also delineating the controllability limits under high C-rates to support coordinated optimization of thermal design and control strategies. The added 2C experimental validation further supports the reliability of extrapolating the model to predict the thermal-regulation trend under 3C high-rate operation. However, as the C-rate increases, the heat-generation intensity and internal temperature gradient of the battery increase significantly, and the results in practical engineering applications may be affected by factors such as the battery heat-generation model, SOC, internal resistance parameters, contact thermal resistance, thermophysical properties, and cold-plate boundary conditions. Therefore, the 3C simulation results are mainly used to predict the thermal-regulation trend of the BTSMS under high-rate conditions and to guide the optimization of CP set temperature and coolant flow rate, rather than being regarded as absolutely precise quantitative engineering predictions.

The superior thermal-management performance can be attributed to the synergistic mechanism between the dual-sided CA layers and CP. The CA layers accelerate temperature field homogenization, while the absorbed heat is efficiently dissipated through the circulating coolant inside CP. Such thermal-management capability ensures stable dissipation of the internal heat generated during high-rate charge and discharge of lithium batteries in the takeoff and landing phases of electric aircraft, thereby enhancing overall safety.

### Thermal-Safety Performance of BTSMS

**Fig. 6:**
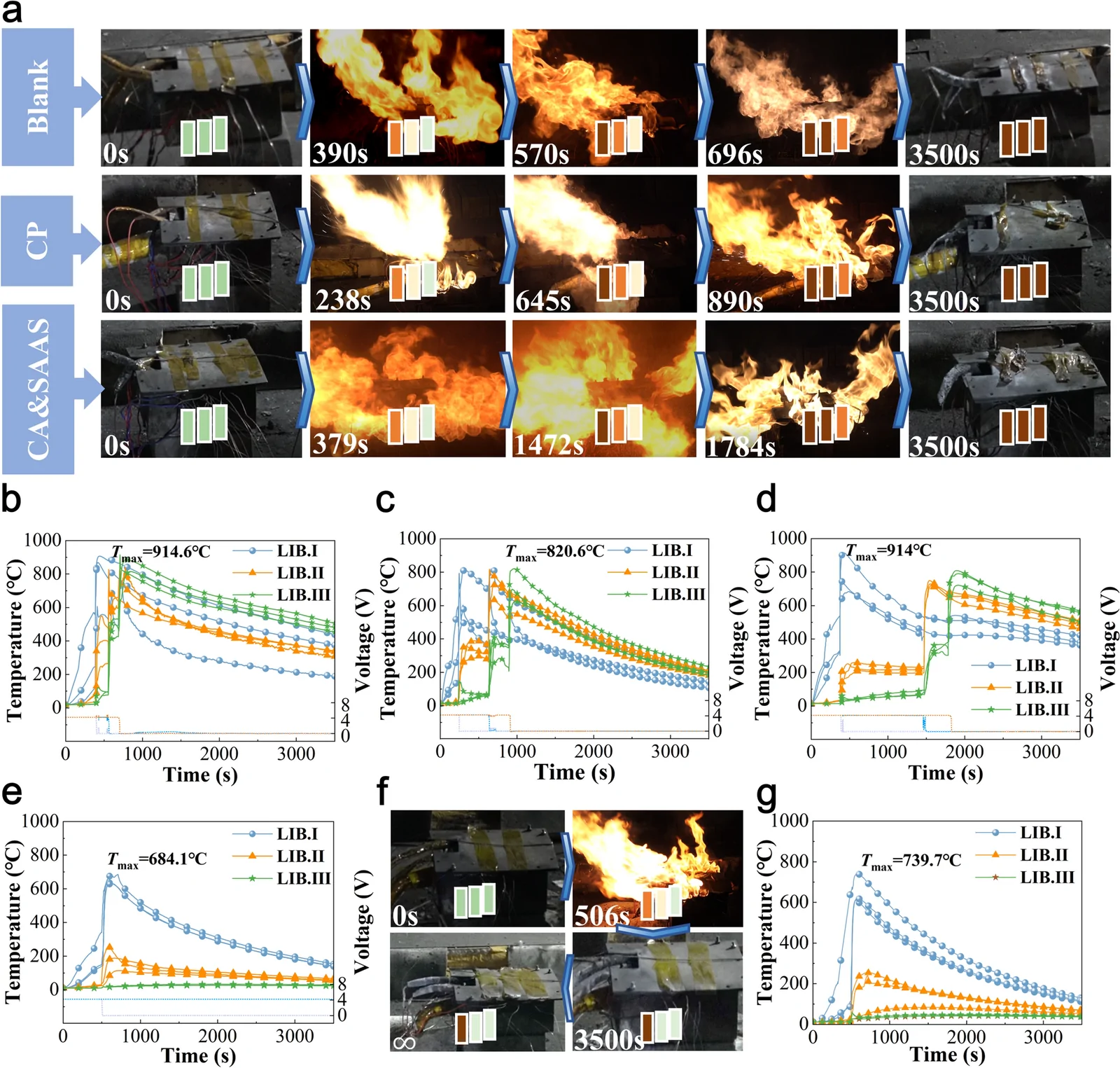
Thermal-safety performance tests. **a **Experimental phenomena of the three control groups. The TR temperature evolution curves of **b** Blank, **c** CP and **d** CA&SAAS. **e** TR temperature evolution curves of BTSMS. **f** TR behaviors of LIBs in BTSMS. **g** Repeated experiments of BTSMS

To evaluate the capability of the BTSMS to suppress thermal runaway propagation (TRP) in electric-aircraft battery packs operating in confined compartments, TR mitigation tests were performed on a three-cell module inside a full-scale aircraft environmental simulation chamber. To further distinguish the functional contributions of CP and CA&SAAS in the BTSMS and exclude the confounding effects introduced by intercell-gap geometry and contact thermal resistance, five comparative configurations were designed: Blank (without CP and CA&SAAS), CP, CA&SAAS, CP+MF, and BTSMS. In the CP+MF group, a needle-punched mullite-fiber felt with the same thickness as CA&SAAS was used as an inert spacer to determine whether the increased intercell spacing and contact thermal resistance alone are sufficient to block TRP. Figure 6a presents the typical experimental phenomena of the Blank, CP, and CA&SAAS groups, while the temperature-rise curve and experimental phenomena of the CP+MF group are shown in Figs. S16 and S17.

The temperature evolution of the Blank group is shown in Fig. 6b. After TR was triggered in LIB. I, the surface temperature of LIB. II rapidly increased from room temperature to 546.8 °C within 74 s, followed by TR after 106 s. Subsequently, LIB. III underwent TR after an additional 126 s, indicating strong intercell thermal coupling and rapid TRP in the confined module without thermal protection. When only CP was used (Fig. 6c), LIB. I underwent TR at 407 s, followed by TR in LIB. II and then in LIB. III after another 245 s. Although CP removed part of the heat and reduced the peak temperature of LIB. I during TR from 914.6 °C in the Blank group to 820.6 °C, it could not effectively suppress intercell thermal radiation or air convection. Therefore, CP alone can only delay TRP rather than block it. When CA&SAAS was applied alone, the TRP time from LIB. I to LIB. II was extended to 1093 s (Fig. 6d), demonstrating the pronounced transverse thermal-barrier effect of CA&SAAS. However, in the absence of an active heat-removal pathway, the accumulated heat in the confined space could not be dissipated in time, eventually leading to TR in the adjacent cell.

Furthermore, the CP+MF group was introduced to evaluate the effect of an equivalent-thickness inert spacer on TRP. In this group, after LIB. I underwent TR, its surface peak temperature reached 768.8 °C, while the surface temperature of LIB. II increased to 400 °C. After 935 s, the safety valve of LIB. II ruptured, followed by TR. Owing to the continuous heat accumulation released from the first two runaway cells in the confined space, LIB. III underwent TR 192 s after LIB. II, with a surface peak temperature of 847 °C. These results indicate that the increased intercell spacing and contact thermal resistance introduced by the equivalent-thickness needle-punched mullite-fiber felt can delay TRP to some extent, but are still insufficient to achieve effective TRP interruption. This is mainly because MF functions only as a spacer and passive thermal barrier; it lacks the synergistic suppression of gas-phase and radiative heat transfer provided by the nanoporous Si/Al aerogel network and does not possess the heat-redistribution and heat-removal capability formed by the coupling between the CA skins and CP.

Notably, when the BTSMS was used, TR in LIB. I did not trigger TR in LIB. II, demonstrating effective interruption of TRP (Fig. 6e-f). Meanwhile, the peak surface temperature of the battery was reduced to 684.1 °C, which was 230.5, 136.5, 229.9, and 84.7 °C lower than those of the Blank (914.6 °C), CP (820.6 °C), CA&SAAS (914.0 °C), and CP+MF (768.8 °C) groups, respectively. Figure S17 shows the three-dimensional temperature-field evolution during TR under different configurations. In particular, in the BTSMS case, heat did not continue to propagate toward LIB. II or LIB. III over time, further confirming the effective suppression of TRP. These results demonstrate that the TRP-blocking capability of the BTSMS is not simply derived from the geometric spacing or contact thermal resistance introduced by the 2-mm interlayer, but mainly originates from the functional thermal barrier of CA&SAAS and the synergistic heat dissipation through CA–CP coupling. Specifically, the SAAS core weakens transverse thermal coupling between adjacent cells, the CA skins promote in-plane spreading of residual heat, and the CP provides a continuous heat-removal pathway, collectively enabling effective TRP interruption.

In addition, to verify the blocking stability and reproducibility of the system, CA&SAAS samples that had already undergone one TR-blocking test were further used for repeated validation. The surface temperature-rise curve is shown in Fig. 6g, confirming that the system can successfully block TRP again and further demonstrating the reliability of the BTSMS for battery thermal-safety protection in confined spaces.

The microstructure change of CA&SAAS after TRP suppression is shown in Fig. S19. SEM revealed that its pore structure only underwent slight contraction after the TRP was blocked. To further verify its thermal insulation performance, XRD analysis was performed. As shown in Fig. S20, after TR, by comparing the XRD patterns of CA&SAAS before TR and after TR, the peak positions and overall profiles remain essentially unchanged: the broad halo associated with amorphous SiO_2_ is retained, and the characteristic reflections assigned to the mullite-fiber framework (PDF#15-0776) show no discernible shift or disappearance. These results indicate a stable phase assemblage and good high-temperature structural robustness after TR exposure. Notably, the reflection near 44.65° becomes more pronounced after TR; this position overlaps with framework-related peaks (e.g., mullite/Al-containing phases), and the intensity increase is more plausibly attributed to the deposition of cell-ejected residues on the CA&SAAS surface (potentially including Ni) rather than the formation of new crystalline phases in the CA&SAAS matrix [66].

The aforementioned experimental observations demonstrate that both CP and CA&SAAS exhibit outstanding capability in mitigating intercell heat transfer. Specifically, CP enables rapid heat dissipation via efficient coolant circulation, whereas the CA&SAAS insulation architecture suppresses heat conduction by introducing low-thermal-conductivity (0.0165 Wm^⁻1^K^⁻1^) pathways. These strategies correspond to active cooling and passive insulation, respectively, thereby alleviating localized temperature rise and limiting heat propagation to adjacent cells. Compared with the Blank (914.6 °C), CP (820.6 °C), and CA&SAAS (914.0 °C) configurations, BTSMS markedly reduces the peak temperature during TR to 684.1 °C; even in the repeated trial, the BTSMS peak temperature remains as low as 739.7 °C. This pronounced mitigation substantially decreases the thermal impact and associated secondary hazards imposed on the surrounding compartment during TR of electric-aircraft lithium-ion battery packs, thereby further enhancing overall aircraft thermal safety.

## Conclusions

In this work, a lightweight sandwich dual-network aerogel (CA&SAAS) was fabricated through in situ deposition and integrated supercritical drying, and a battery thermal-safety management system (BTSMS) was further constructed by coupling CA&SAAS with a cold plate for electric-aircraft battery packs in confined compartments. CA&SAAS exhibited a bulk density of 0.275 gcm^⁻3^ and a specific surface area of 626.37 m^2^g^⁻1^, confirming its lightweight porous feature. The SAAS core retained a low thermal conductivity of 0.0652 Wm^⁻1^K^⁻1^ at 1000 °C, while the full CA&SAAS composite showed a room-temperature thermal conductivity of 0.019 Wm^⁻1^ K^⁻1^. After 1000 compression cycles at 30% strain, SAAS still maintained thermal conductivities of 0.01622 and 0.0664 Wm^⁻1^K^⁻1^ at room temperature and 1000 °C, respectively, demonstrating stable mechanical–thermal insulation reliability. During four 1C charge–discharge cycles, BTSMS limited *T*_max_/*ΔT*_max_ to 34.9/1.8 °C, reducing peak temperature and temperature nonuniformity by 46.5% and 56.1%, respectively. Predictive simulations provide engineering-relevant CP design parameters and indicate that *T*_max_ can be constrained to around 45 °C under 3C operation. TR tests in a full-scale aircraft environmental chamber further confirm that the BTSMS effectively interrupts TRP in a confined three-cell module, demonstrating its application potential for thermal-safety management in electric-aircraft power lithium-ion battery packs.

## Supplementary Information

Below is the link to the electronic supplementary material.

Supplementary file1 (DOCX 4997 KB)

## List of Symbols

*T*: Temperature(°C)
*T*_max_: Maximum temperature(°C)
*ΔT*: Temperature difference(°C)
*ΔT*_max_: Maximum temperature difference(°C)
C: Charge/discharge rate(/)
L: Battery length(mm)
W: Battery width(mm)
He: Battery height(mm)
t: Time(s)
λ: Thermal conductivity(W·m⁻^1^·K⁻^1^)
G′: Storage modulus(kPa)
G″: Loss modulus(kPa)
ζ: Damping metric(/)
q″: Volumetric heat-generation rate(W·m⁻^3^)
a: Coordinates along the thickness direction(m)
d: Thickness(m)
d_CA,1_/d_CA,2_: Thickness of carbon aerogel on both sides(m)
d_SAAS_: Thickness of SAAS(m)
m/n: M/n represents any layer in CA&SAAS(/)
ρ_m_: The density of layer m(kg·m⁻^3^）
c_m_: The specific heat capacity of layer m(J·kg⁻^1^·K⁻^1^)
*T*_m_/ *T*_n_: The temperature of layer m/n(K)
λ_m__,eff_: Effective thermal conductivity(W·m⁻^1^·K⁻^1^)
λ_m__,eff_ (*T*): The effective thermal conductivity of layer m, which may vary with temperature.(W·m⁻^1^·K⁻^1^)
λ_solid__,m_: Solid-phase thermal conductivity(W·m⁻^1^·K⁻^1^)
λ_gas__,m_: Gas-phase thermal conductivity(W·m⁻^1^·K⁻^1^)
λ_rad__,m_: Radiative thermal conductivity(W·m⁻^1^·K⁻^1^)
λ_gas__,0_: The free-gas thermal conductivity(W·m⁻^1^·K⁻^1^)
a_int_: The interface position(m)
a_int_^+^: The right side of the interface(m)
a_int_^−^: The left side of the interface(m)
D: Pore size(m)
l: The molecular mean free path(m)
Kn: Knudsen number(/)
Φ: The open porosity(/)
β: The energy accommodation at the gas–solid interface(/)
β_R_: The Rosseland mean extinction coefficient(m^−^^1^)
β': The mean extinction coefficient(m^3^)
q: The volumetric heat-generation rate(W·m^−^^3^)
I: Current(A)
V: The cell volume(m^3^)
E: Open-circuit voltage/electromotive force(V)
U: Terminal operating voltage(V)
R_j_: Ohmic resistance(Ω)
R_p_: Polarization resistance(Ω)
ρ_l_: The fluid density(kg·m⁻^3^)
u: The fluid velocity(m·s⁻^1^)
p: Pressure(Pa)
${\tau}_{ij}$: The viscous shear stress tensor(Pa)
${\tau}_{ij}^{R}$: Reynolds stress tensor(Pa)
${\delta}_{ij}$: Kronecker delta; ${\delta}_{ij}=1$ when i=j, otherwise ${\delta}_{ij}=0$
S_i_: The mass-distributed external force per unit mass(N·kg⁻^1^)
H: The total enthalpy content(J·kg⁻^1^)
ε: The turbulent dissipation(m^2^·s⁻^3^)
h: The thermal enthalpy(J·kg⁻^1^)
x_i_, x_j_, x_k_: Space coordinates, x/y/z directions(/)
i, j, k: Tensor indices in the Cartesian coordinate system; $i,j,k=\mathrm{1,2},3$
u_i_, u_j_, u_k_: Velocity components in the i, j, and k directions(m·s⁻^1^)
Q_H_: Volumetric heat source term in the energy equation(W·m^−^^3^)
*μ*: The dynamic viscosity coefficient(Pa·s)
*μ*_t_: The turbulent eddy viscosity coefficient(Pa·s)
k: The turbulent kinetic energy(m^2^·s⁻^2^)
C_1ε_, C_2ε_, σ_k_, σ_ε_: The model constants(/)
S_k_, S_ε_: The defined source terms(/)

## Greek symbols

*γ*-Al_2_O_3_: Gamma phase
*δ*-Al_2_O_3_: Delta phase
*α*-Al_2_O_3_: Alpha phase
*Δ*: Difference

## Subscripts

max: Maximum value
x: X-direction
y: Y-direction
z: Z-direction

## Superscripts

R: Reynolds stress

## Acronyms

CA&SAAS: Carbon–Si/Al–Carbon dual-network aerogel
CP: Cold plate
BTSMS: Battery thermal-safety management system
SAAS: Silica–alumina aerogel sheet
CA: Carbon aerogel
MF: Mullite fiber
TR: Thermal runaway
TRP: Thermal runaway propagation
eVTOL: Electric vertical takeoff and landing
PCM: Phase-change material
TEOS: Tetraethyl orthosilicate
EtOH: Ethanol
NH_3_·H_2_O: Ammonia solution
CS: Composite sheet
RF: Resorcinol–furfural
SEM: Scanning electron microscopy
BET: Brunauer–Emmett–Teller
XRD: X-ray diffraction
XPS: X-ray photoelectron spectroscopy
FTIR: Fourier transform infrared spectroscopy
DMA: Dynamic mechanical analysis
TGA: Thermogravimetric analysis
NCM: Lithium nickel cobalt manganese oxide
LIB.: Lithium battery
SOC: State of charge
CC-CV: Constant current–constant voltage
CC: Constant current
CV: Constant voltage
HPPC: Hybrid pulse power characterization
DCR: Direct current resistance
OCV: Open-circuit voltage
CFD: Computational fluid dynamic
EDS: Energy-dispersive spectroscopy
STI: Composite thermal insulation board
PW/HGA: Paraffin wax/hybrid graphene aerogel
PEA: Polyethylene aerogel
SAFAs: Silica-alumina nanofibrous aerogels
PDMS/PE: Poly(dimethylsiloxane) with polyethylene
PVDF-HFP: Poly(vinylidene fluoride-hexafluoropropylene)
Blank: Blank control group in the experiment
CP + MF: Cold plate + mullite fiber
LIB.Ⅰ, LIB.Ⅱ, LIB.Ⅲ: The first battery, the second battery, and the third battery
