# Supplementary material for: Dual-Network Aerogel-Based Thermal-Safety Management System Design for Electric-Aircraft Battery Packs: Efficient Heat Management and Runaway Protection
Source: Nanomicro Lett. 2026 Jul 21;18:447. doi: 10.1007/s40820-026-02296-4 (PMC13388894; doi:10.1007/s40820-026-02296-4)
Supplement: Supplementary file 1 — Supplementary file1 (DOCX 4997 KB) [file 40820_2026_2296_MOESM1_ESM.docx]

Supporting Information for

**Dual-Network Aerogel–Based Thermal Safety Management System Design for Electric-Aircraft Battery Packs: Efficient Heat Management and Runaway Protection**

Jie Yang^1^, Yueyue Xiao^1^, Mingyuan Yan^2^, Xu Huang^1^, Longlong Li^1^, Zhongxin Zhang^3^, Xudong Cheng^1^, Heping Zhang^1,^ * and Yuelei Pan^1,^ *

^1^ State Key Laboratory of Fire Science, University of Science and Technology of China, Hefei 230027, P. R. China

^2^ School of Public Security and Emergency Management, Anhui University of Science and Technology, Hefei 231131, P. R. China

^3^ Institute of Advanced Technology, University of Science and Technology of China，Hefei 230027, P. R. China

* Corresponding authors. E-mail: [zhanghp@ustc.edu.cn](mailto:zhanghp@ustc.edu.cn) (Heping Zhang); [panyl@ustc.edu.cn](mailto:panyl@ustc.edu.cn) (Yuelei Pan)

**S1 Supplementary Text**

***S1.1 Numerical Modeling of Conjugate Heat Transfer***

A conjugate heat-transfer model was developed in STAR-CCM+ (Version 2410), where heat exchange across solid–fluid interfaces and between dissimilar material domains was implemented via a multiphysics coupling framework. The fluid domain was governed by the transient Navier–Stokes equations, which account for transient, convective, viscous-diffusion, and source terms, representing the mathematical forms of mass, momentum, and energy conservation. The conservative form can be written as:

$\frac{\text{∂}\text{ρ}_{\text{l}}}{\text{∂t}}\text{ + }\frac{\text{∂}}{\text{∂}\text{x}_{\text{i}}}\text{ (ρ }\text{u}_{\text{i}}\text{) = 0}$ (S1)

$\frac{\text{∂ρ}\text{u}_{\text{i}}}{\text{∂t}}\text{+}\frac{\text{∂}}{\text{∂}\text{x}_{\text{j}}}\left( \text{ρ}\text{u}_{\text{i}}\text{u}_{\text{j}} \right)\text{+}\frac{\text{∂p}}{\text{∂}\text{x}_{\text{i}}}\text{=}\frac{\text{∂}}{\text{∂}\text{x}_{\text{j}}}\left( \text{τ}_{\text{ij}}\text{+}\text{τ}_{\text{ij}}^{\text{R}} \right)\text{+}\text{S}_{\text{i}}\text{ i=1,2,3}$ (S2)

$\frac{\text{∂ρH}}{\text{∂t}}\text{+}\frac{\text{∂ρ}\text{u}_{\text{i}}\text{H}}{\text{∂}\text{x}_{\text{i}}}\text{=}\frac{\text{∂}}{\text{∂}\text{x}_{\text{j}}}\left[ \text{u}_{\text{j}}\left( \text{τ}_{\text{ij}}\text{+}\text{τ}_{\text{ij}}^{\text{R}} \right)\text{+}\text{q}_{\text{i}} \right]\text{+}\frac{\text{∂p}}{\text{∂}_{\text{t}}}\text{-}\text{τ}_{\text{ij}}^{\text{R}}\frac{\text{∂}\text{u}_{\text{i}}}{\text{∂}\text{x}_{\text{j}}}\text{+ρε+}\text{S}_{\text{i}}\text{u}_{\text{i}}\text{+}\text{Q}_{\text{H}}$ (S3)

$\text{H=h+}\frac{\text{u}^{\text{2}}}{\text{2}}$ (S4)

Where $\text{ρ}_{\text{l}}$ is the fluid density, u is the fluid velocity, τ_ij_ is the viscous shear stress tensor, S_i_ is a mass-distributed external force per unit mass, H is the total enthalpy content, q_i_ is the diffusive heat flux. $\text{ε}$ is the turbulent dissipation, h is the thermal enthalpy.

Most fluid flows in battery cooling plate are turbulent. To simulate and predict turbulent flows, time-averaged effects of the flow turbulence on the flow parameters are considered. To close the equations, it mostly employs transport equations for the turbulent kinetic energy and its dissipation rate, the so-called k-ε model. The Reynolds-stress tensor has the following form as Boussinesq assumption:

$\text{τ}_{\text{ij}}^{\text{R}}\text{=}\text{μ}_{\text{t}}\left( \frac{\text{∂}\text{u}_{\text{i}}}{\text{∂}\text{x}_{\text{j}}}\text{+}\frac{\text{∂}\text{u}_{\text{j}}}{\text{∂}\text{x}_{\text{i}}}\text{-}\frac{\text{2}}{\text{3}}\text{δ}_{\text{ij}}\frac{\text{∂}\text{u}_{\text{k}}}{\text{∂}\text{x}_{\text{k}}} \right)\text{-}\frac{\text{2}}{\text{3}}\text{ρkδ}_{\text{ij}}$ (S5)

Here μ_t_ is the dynamic viscosity coefficient, μ_t_ is the turbulent eddy viscosity coefficient and k is the turbulent kinetic energy. Two transport equations are used to describe the turbulent kinetic energy and dissipation:

$\frac{\text{∂ρk}}{\text{∂t}}\text{+}\frac{\text{∂}}{\text{∂}\text{x}_{\text{i}}}\left( \text{ρ}\text{u}_{\text{i}}\text{k} \right)\text{=}\frac{\text{∂}}{\text{∂}\text{x}_{\text{i}}}\left[ \left( \text{μ+}\frac{\text{u}_{\text{t}}}{\text{σ}_{\text{k}}} \right)\frac{\text{∂k}}{\text{∂}\text{x}_{\text{i}}} \right]\text{+}\text{u}_{\text{t}}\left( \frac{\text{∂}\text{u}_{\text{i}}}{\text{∂}\text{x}_{\text{j}}}\text{+}\frac{\text{∂}\text{u}_{\text{j}}}{\text{∂}\text{x}_{\text{i}}} \right)\frac{\text{∂}\text{u}_{\text{j}}}{\text{∂}\text{x}_{\text{i}}}\text{-ρε+}\text{S}_{\text{k}}$ (S6)

$\frac{\text{∂}}{\text{∂t}}\left( \text{ρε} \right)\text{+}\frac{\text{∂}}{\text{∂}\text{x}_{\text{i}}}\left( \text{ρε}\text{u}_{\text{i}} \right)\text{=}\frac{\text{∂}}{\text{∂}\text{x}_{\text{i}}}\left[ \left( \text{μ+}\frac{\text{u}_{\text{t}}}{\text{σ}_{\text{ε}}} \right)\frac{\text{∂ε}}{\text{∂}\text{x}_{\text{i}}} \right]\text{+}{\text{C}_{\text{1ε}}\frac{\text{ε}}{\text{k}}\text{u}}_{\text{t}}\left( \frac{\text{∂}\text{u}_{\text{i}}}{\text{∂}\text{x}_{\text{j}}}\text{+}\frac{\text{∂}\text{u}_{\text{j}}}{\text{∂}\text{x}_{\text{i}}} \right)\frac{\text{∂}\text{u}_{\text{j}}}{\text{∂}\text{x}_{\text{i}}}\text{-}\text{C}_{\text{2ε}}\text{ρ}\frac{\text{ε}^{\text{2}}}{\text{k}}\text{+}\text{S}_{\text{ε}}$ (S7)

Where C_1ε_, C_2ε_, σ_k_, σ_ε_ are model constants, C_1ε_ = 1.44, C_2ε_ = 1.92, σ_k_ = 1.0, σ_ε_ = 1.3; S_k_ and S_ε_ are defined source terms. [S1]

***S1.2 Comparison of In-Plane Heat-Spreading Capability between SAAS and CA&SAAS***

To further verify the enhancement of rapid in-plane heat diffusion induced by the carbon aerogel (CA) skins, the in-plane thermal conductivities of SAAS and CA&SAAS were measured and compared. The in-plane thermal conductivity of SAAS is 0.04 W·m⁻¹·K⁻¹, whereas that of CA&SAAS increases to 0.20 W·m⁻¹·K⁻¹, approximately five times higher than that of SAAS. Furthermore, localized heating was applied to the center of SAAS and CA&SAAS samples with the same size (10 × 10 cm) using an alcohol lamp, and the temperature-homogenization behavior on the backside during cooling was compared. After the maximum backside temperature reached 100 °C, the temperatures at five surface measurement points gradually converged during the cooling process. After 300 s, the temperature difference among the five measurement points of CA&SAAS decreased to 1.3 °C (Fig. S10), whereas SAAS still maintained a temperature difference of 3.7 °C under the same conditions (Fig. S11). Compared with SAAS, CA&SAAS reduced the residual temperature difference at 300 s by 64.9%, indicating that CA&SAAS can achieve a more uniform surface temperature distribution within the same cooling period.

***S1.3 Input Parameters and Output Variables of the Model***

The input parameters of the numerical model mainly include geometrical parameters, thermophysical properties, heat-source parameters, and initial/boundary conditions. The geometrical parameters include the dimensions of the 58 Ah prismatic cells, the arrangement of the three-cell module, the busbar connection, and the structural dimensions of the liquid cold plate. The thermophysical properties include the equivalent density, specific heat capacity, and orthotropic thermal conductivity of the cells; the properties of the busbars, thermal adhesive, cold plate, external casing, and CA&SAAS; and the density, dynamic viscosity, specific heat capacity, and thermal conductivity of the coolant. The heat-source parameters include the charge/discharge current, SOC variation, DC resistance obtained from HPPC tests, busbar resistance, and the volumetric heat source calculated from the battery heat-generation model. The initial and boundary conditions include the initial ambient temperature, initial SOC, coolant inlet temperature, inlet flow rate, pressure outlet boundary, and convective heat transfer between the battery-pack surface and the ambient environment. The typical outputs of the model include the cell temperature–time curves, coolant inlet/outlet temperature–time curves, and NTC monitoring-point temperature curves. Based on these outputs, the regulation effect of the BTSMS on peak cell temperature and heat-removal capability under different C-rates, cold-plate temperatures, and coolant flow rates can be further evaluated.

***S1.4 Grid-Independence Study***

To ensure the accuracy and reliability of the three-dimensional conjugate heat-transfer simulation, a grid-independence study was conducted for the liquid-cooled battery-pack model. Considering the complex internal geometry of the liquid-cooled battery pack, including thin-walled cooling channels, irregular air gaps, cell tabs, and busbar-connection regions, and given the sensitivity of coolant-side convective heat transfer to the near-wall mesh, a mesh-generation strategy combining progressive boundary-layer optimization and local refinement was adopted. The grid-independence analysis was performed under the 3C limiting operating condition in which the maximum temperature of the whole cell reached 50 °C at the tab region, corresponding to a jelly-roll heat-generation power of 480 W, so as to make the temperature-response differences among different mesh schemes more distinguishable. It should be noted that the maximum temperature *T*_max_ reported for different mesh schemes refers to the maximum temperature of the whole single cell, including the tab region, rather than only the surface temperature of the main cell body.

Four computational meshes with different densities were generated for comparison. The mesh schemes were designed as follows: Grid 1 was the initial coarse mesh without fluid boundary layers; Grid 2 adopted global refinement with three fluid boundary layers; Grid 3 used further global refinement with four fluid boundary layers; and Grid 4 fixed the boundary-layer parameters and applied local refinement in the heat-generation and cooling regions. In the first three mesh schemes, the spatial mesh size was gradually reduced, and the boundary-layer setting at the coolant–solid interface was progressively optimized. After confirming that the four-layer boundary mesh could reasonably capture the near-wall temperature gradient, the fourth mesh scheme maintained the same boundary-layer parameters and further refined the heat-generation and cooling regions to evaluate the influence of spatial discretization error. The calculated results under different mesh schemes are summarized in Table S7.

When the number of cells increased from 490,059 to 961,383, the maximum temperature of the whole single cell changed only from 88.2 °C to 88.1 °C, with a temperature difference of 0.1 °C and a relative deviation below 0.5%. This indicates that further mesh refinement has only a minor influence on the calculation results, and the numerical solution can be regarded as grid independent. Considering both computational accuracy and the cost of subsequent transient cycling simulations, Grid 3 with 490,059 cells was selected as the standard mesh for the following thermal-management simulations.

**S2 Supplementary Figures and Tables**


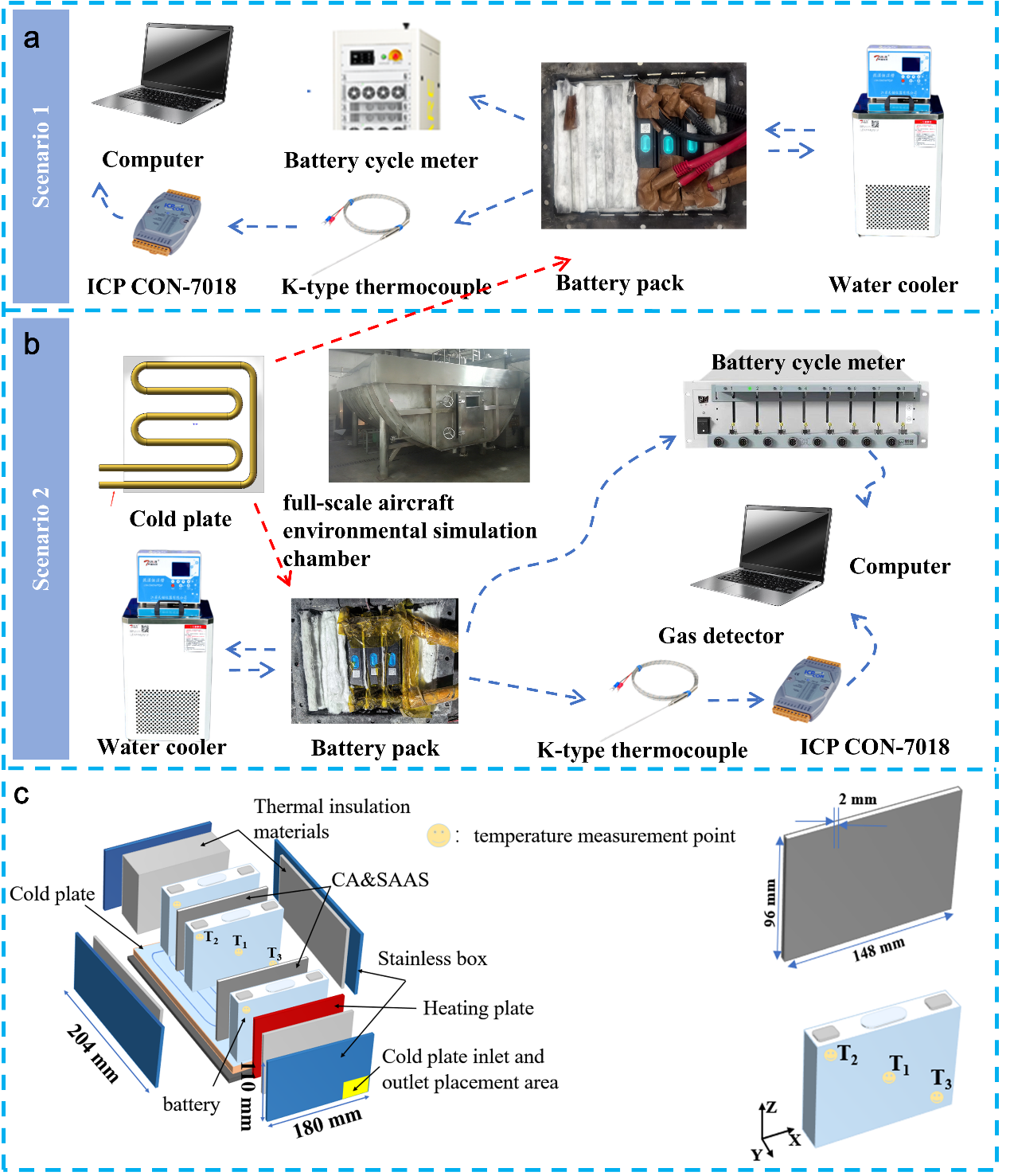


**Fig. S1** The thermal safety management performance test platform; **a** The thermal-management performance test platform; **b** TRP platform; **c** internal module layout and thermocouple positions.


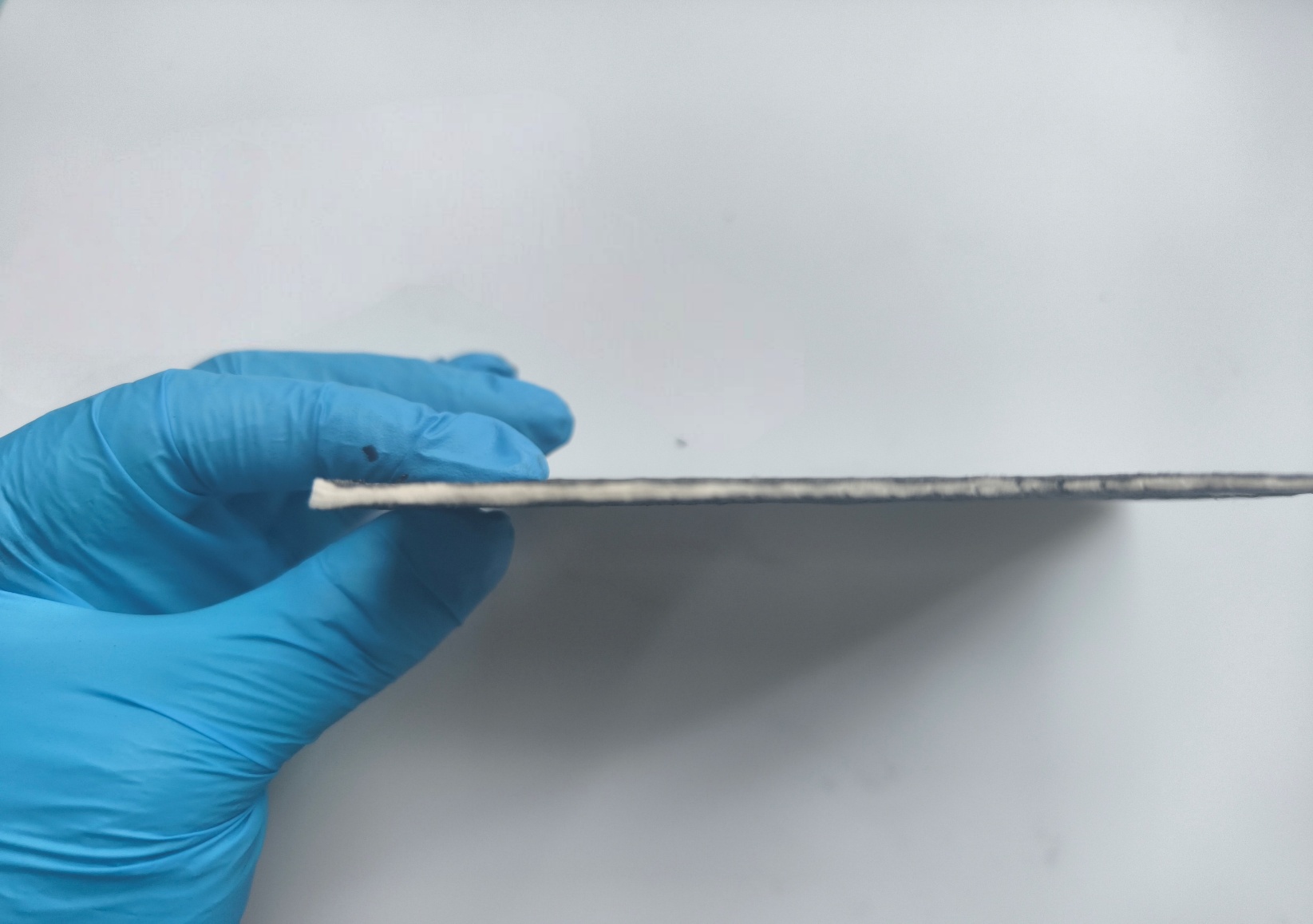


**Fig. S2** Macroscopic schematic illustration of the sandwich structure of CA&SAAS.


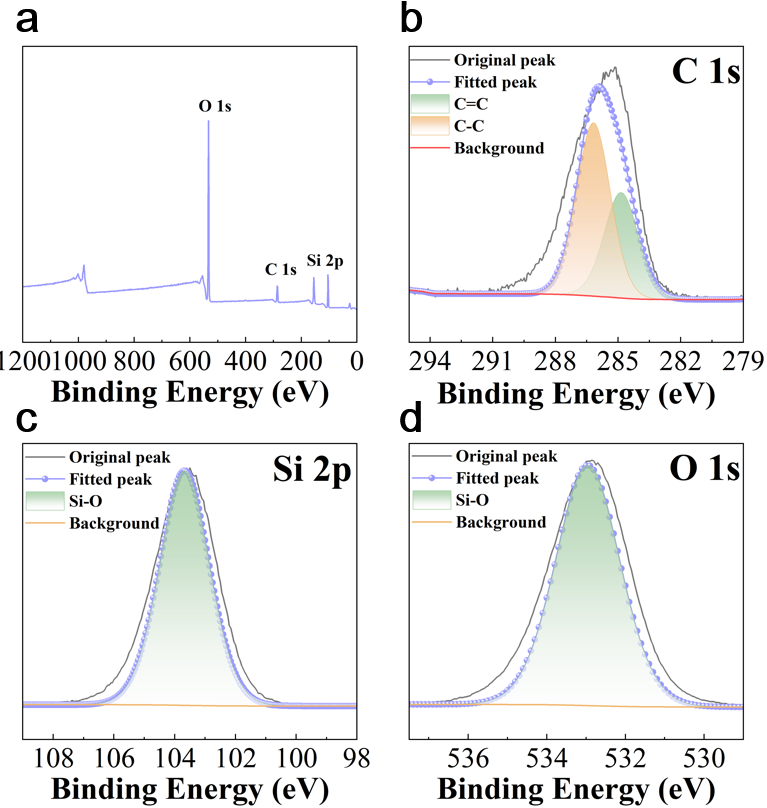


**Fig. S3** XPS of aerogel: **a** Survey; **b** C 1s; **c** Si 2p; **d** O 1s.


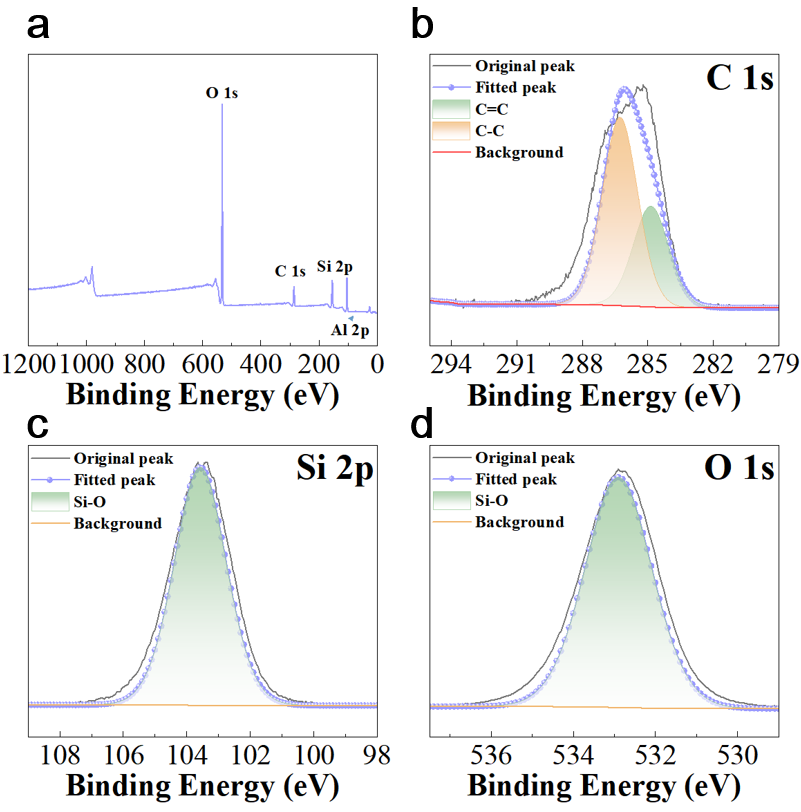


**Fig. S4** XPS of SAAS: **a** Survey; **b** C 1s; **c** Si 2p; **d** O 1s.


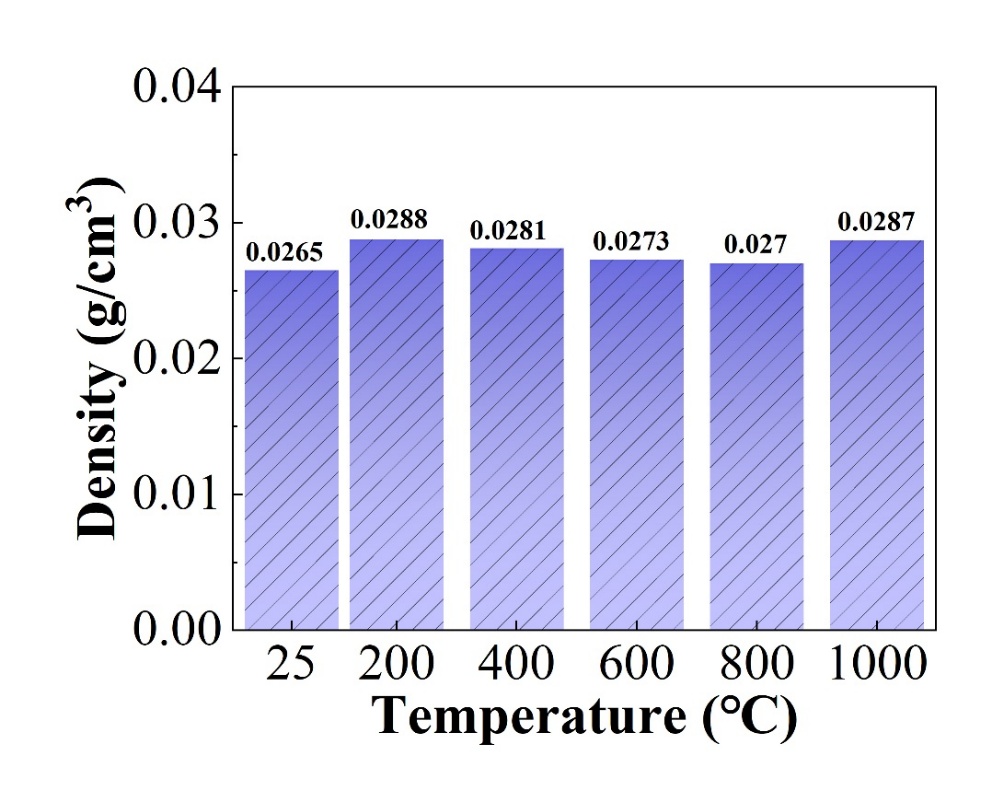


**Fig. S5** Density changes of SAAS after heat treatment at different temperatures


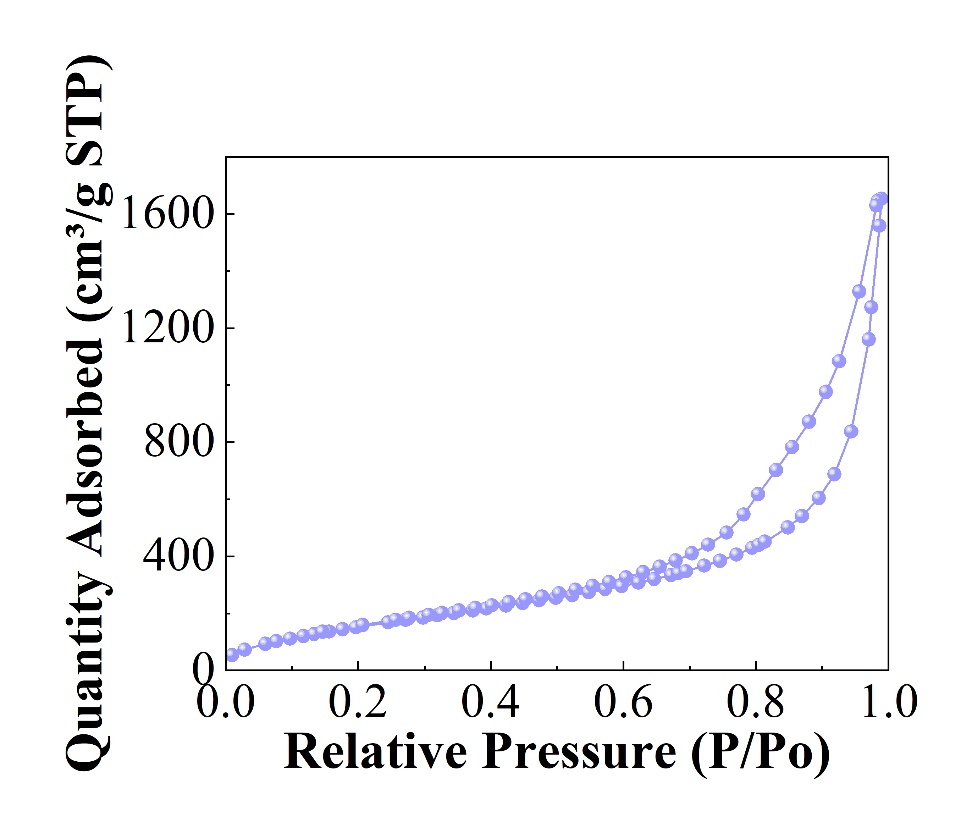


**Fig. S6** Nitrogen adsorption desorption curve of CA&SAAS


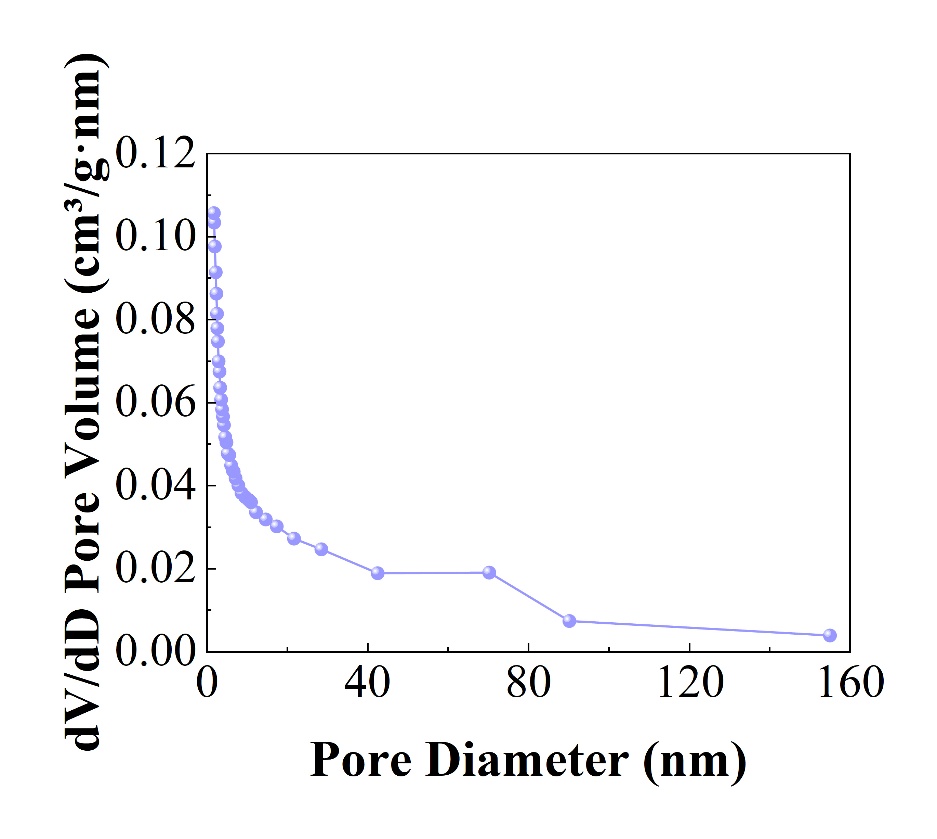


**Fig. S7** Pore size distribution of CA&SAAS


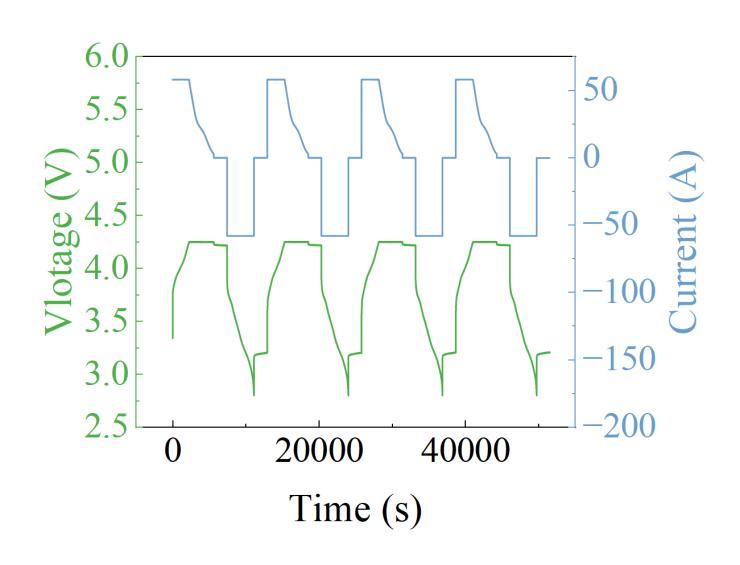


**Fig. S8** Test charge-discharge currents and voltages during the cycles.


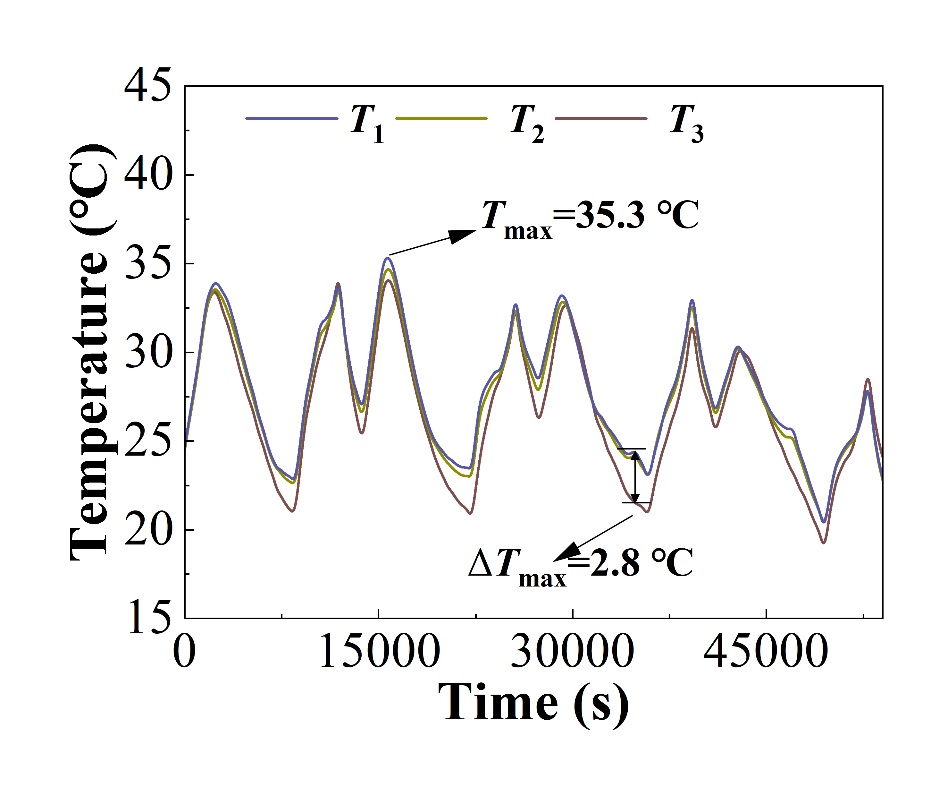


**Fig. S9** Temperature–time curve of the CP+MF group.


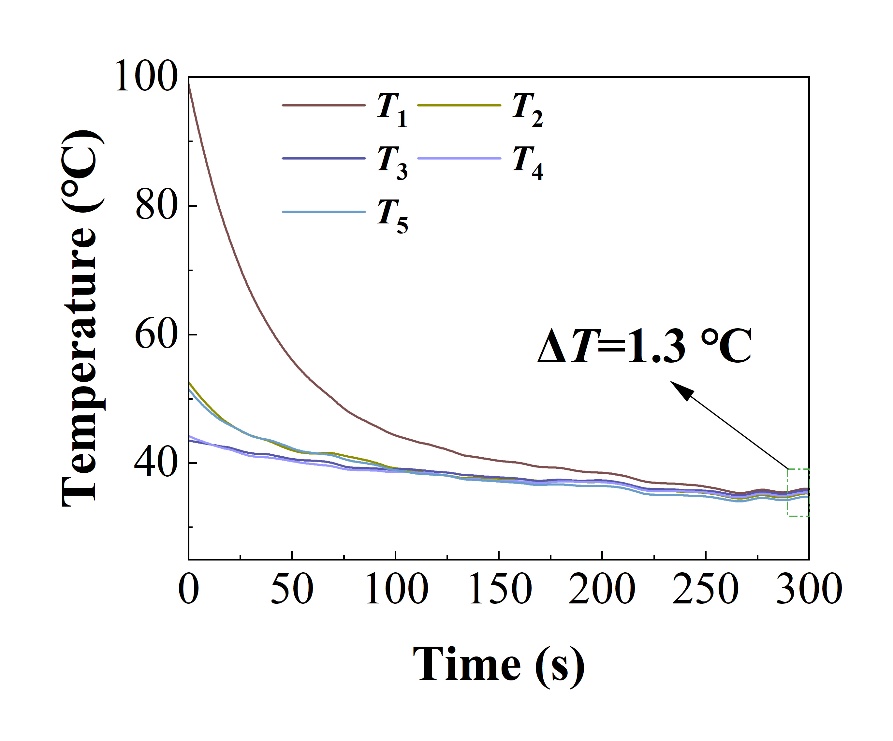


**Fig. S10** Temperature variation curve of CA&SAAS.


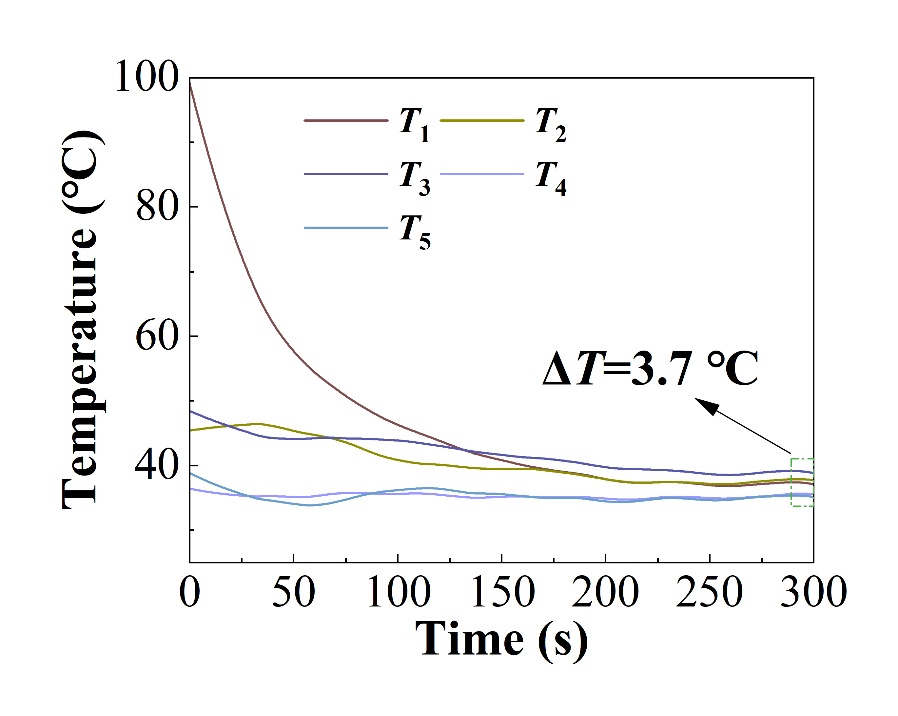


**Fig. S11** Temperature variation curve of SAAS.


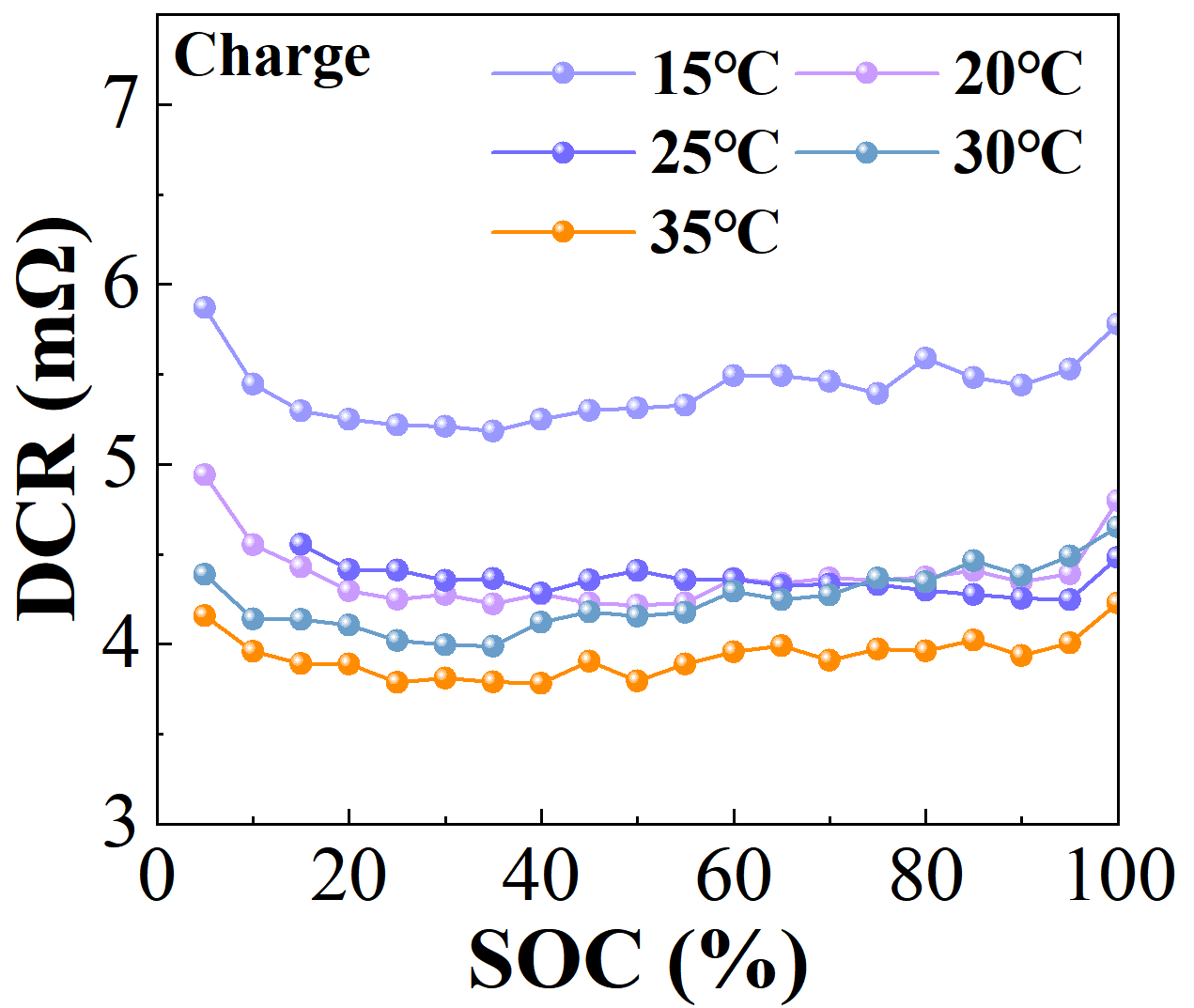


**Fig. S12** Diagram of charging DCR changing with SOC


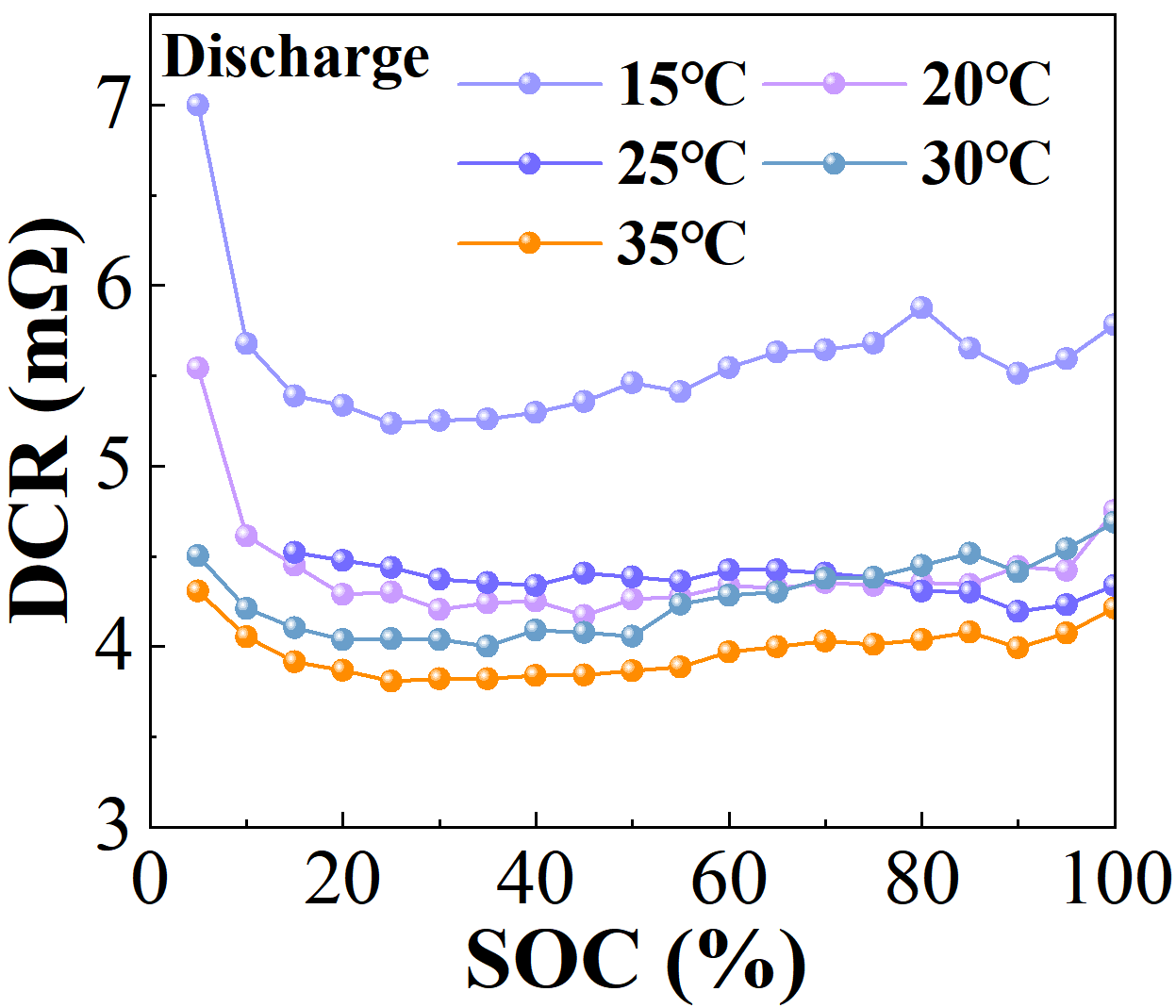


**Fig. S13** Diagram of discharging DCR changing with SOC


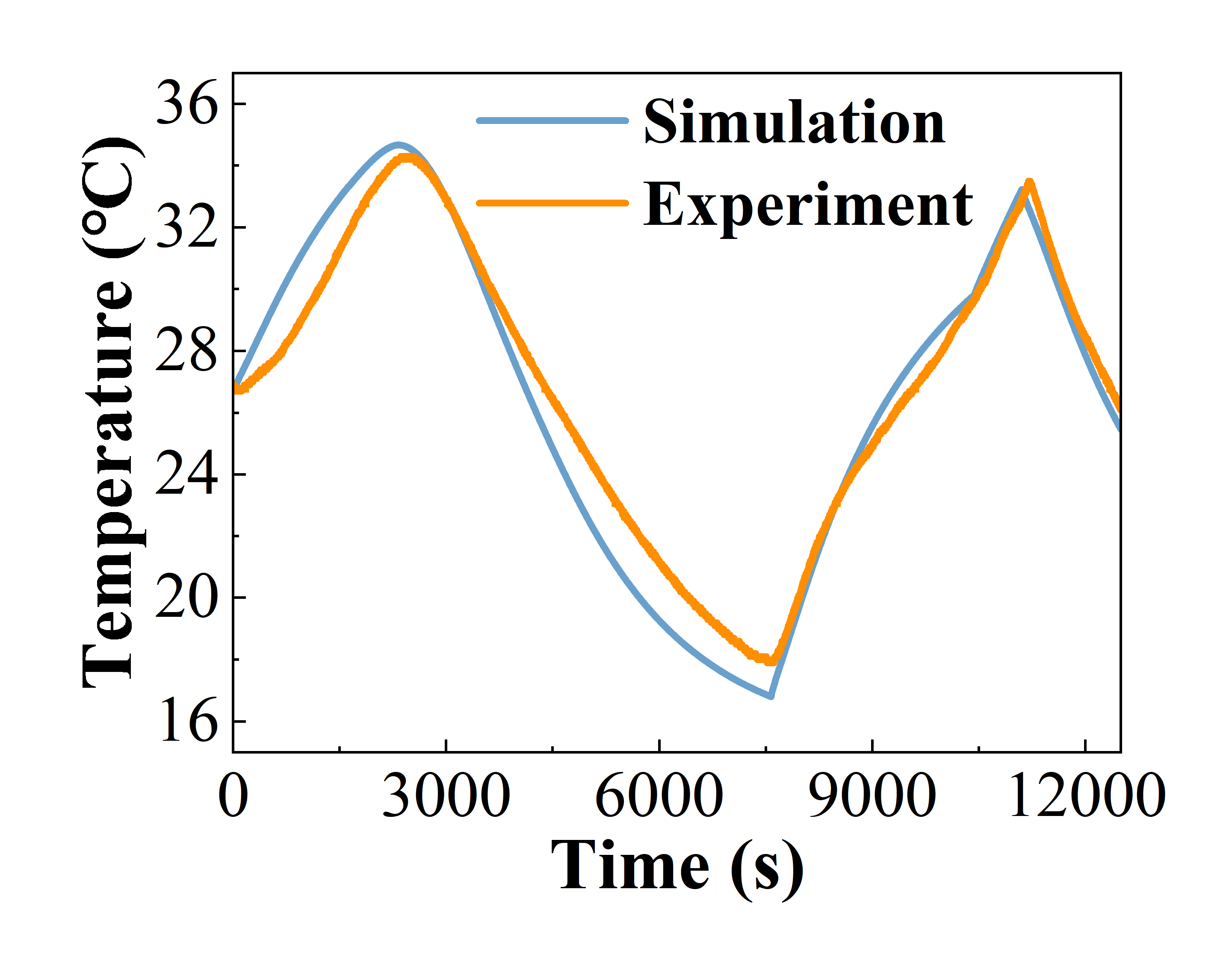


**Fig. S14** Comparison chart of experimental and simulated data under 1C rate charge-discharge conditions


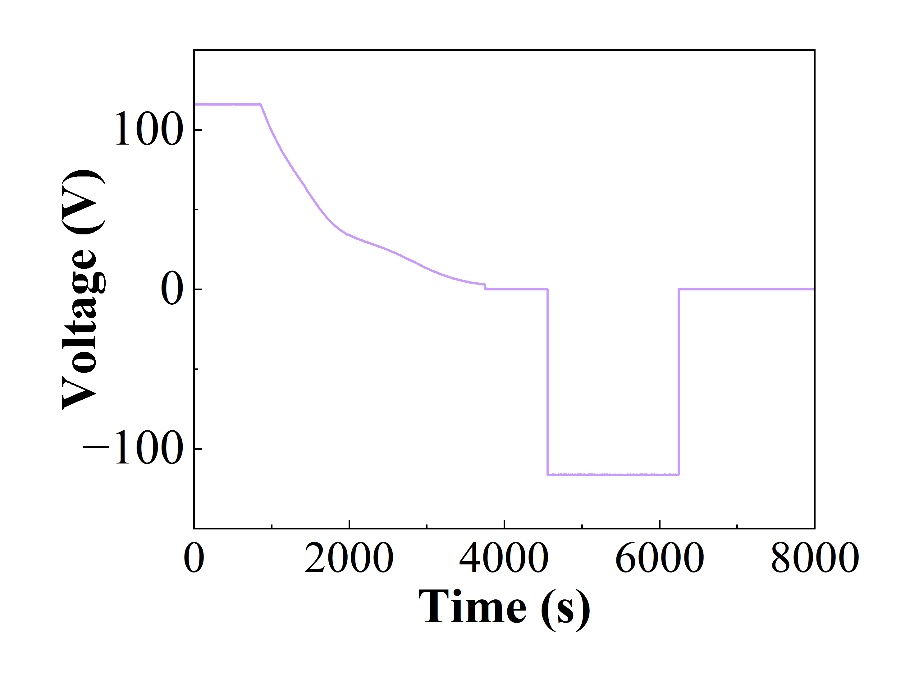


**Fig. S15** Current curve of 2C experimental process


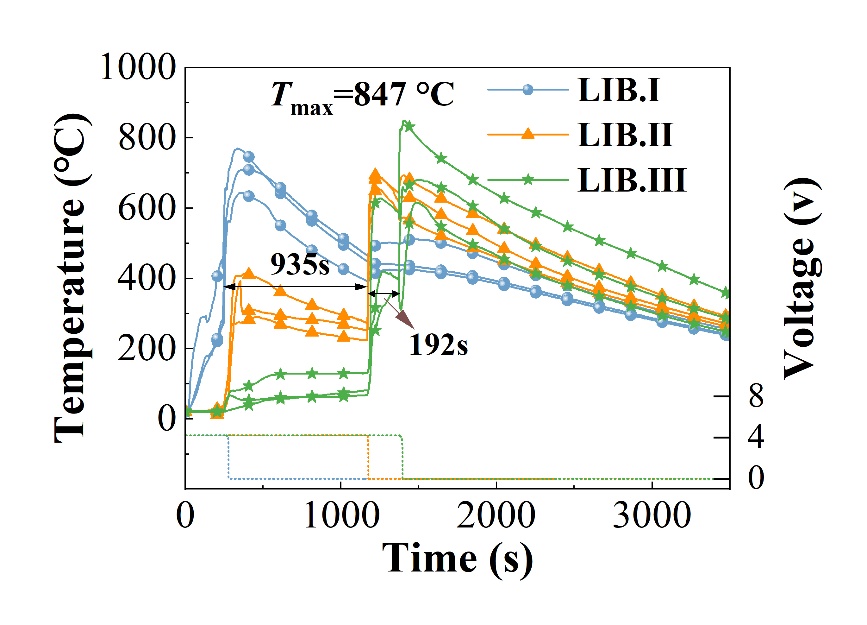


**Fig. S16** Temperature time curve of CP+MF group during TR process


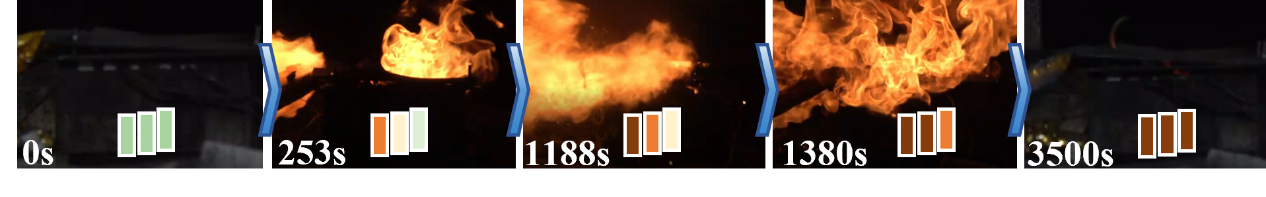


**Fig. S17** CP+MF thermal runaway process diagram


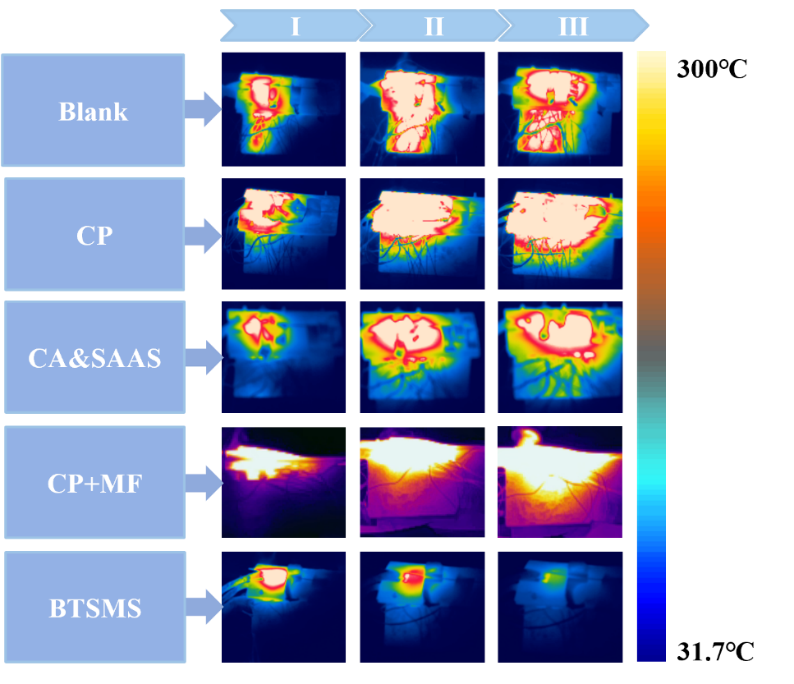


**Fig. S18** the 3D temperature field distribution during TR under different operating conditions.


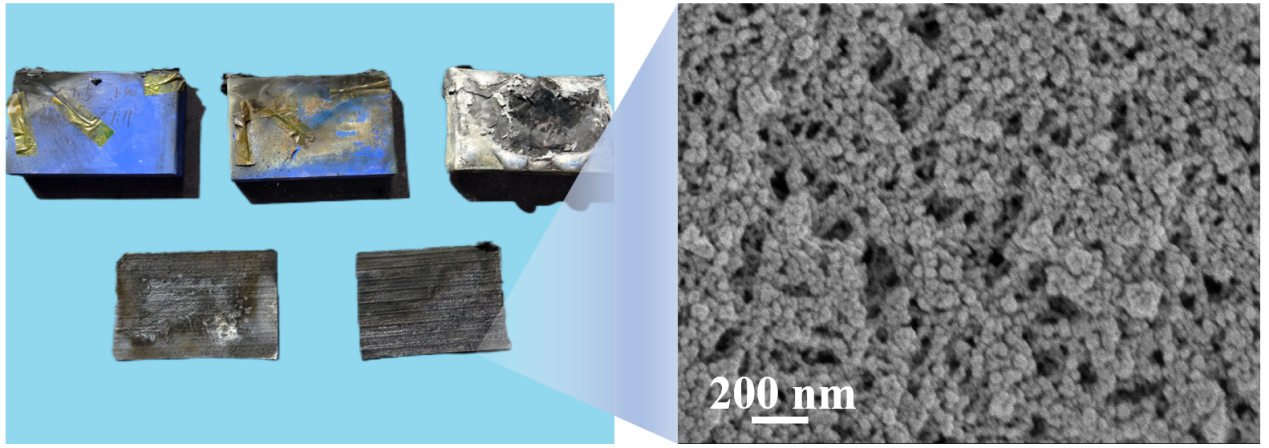


**Fig. S19** Microscopic morphology image of CA&SAAS after TR

**
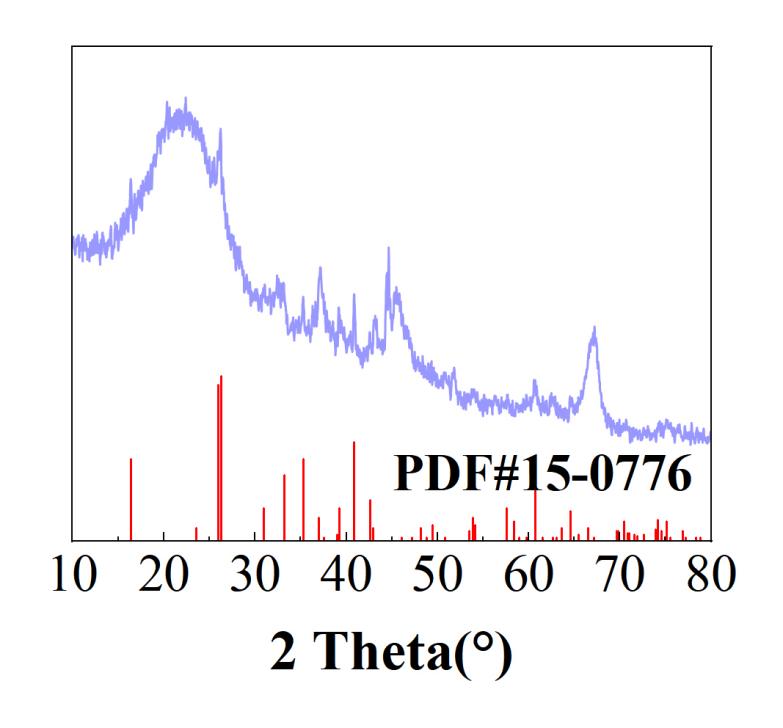
**

**Fig. S20** The XRD of CA&SAAS after TR

**Table S1** The primary parameters of the tested NCM lithium-ion battery.

| Items | Standards | Remarks |
| --- | --- | --- |
| Cathode | Li(Ni_0.8_Co_0.1_Mn_0.1_)O_2_ |  |
| Anode | Graphite |  |
| Electrolyte | Carbonate-based liquid electrolyte (EC/EMC/LiPF6) |  |
| Dimension (length × width× height) | 148.20 ×26.72× 95.21 | With terminal |
| Nominal capacity | 58.0Ah | 1C, 25±2 °C, 2.8-4.25V |
| Nominal voltage | 3.62 | 1C，2.8-4.25V |
| Maximum charging voltage | 4.25V |  |
| Minimum discharge voltage | 2.8V | >0 °C |
| Standard charge/discharge current | 58A | 1C |
| Weight | 860g | Including insulating film |
| SOC | 100% |  |
| Energy | 210.0Wh | 1C, 25±2 °C, 2.8-4.25V |
| Mean value of thermal conductivity | 15-20 | X/Z direction |
|  | 0.5-3 | Y direction |

**Table S2** battery pack parameters

| Items | Remarks |
| --- | --- |
| material | stainless steel |
| Dimension (length × width× height) | 204 mm ×180 mm ×110 mm |
| shell thickness | 5 mm |
| Outer diameter of copper tube | 10 mm |

**Table S3** Cold plate parameters

| Items | Remarks |
| --- | --- |
| material | copper |
| Dimension (length × width× height) | 170 mm ×160 mm ×12 mm |
| Inner diameter of copper tube | 8 mm |
| Outer diameter of copper tube | 10 mm |

**Table S4** Parameters related to cold water circulation machine

| Items | Remarks |
| --- | --- |
| Temperature control range | -10~100 °C |
| Temperature fluctuation range | ±0.05 °C |

**Table S5** Heating plate parameters

| Items | Remarks |
| --- | --- |
| Power | 500 W |
| Dimension (length × width× height) | 148 mm ×98 mm ×2 mm |

**Table S6** The thermocouple information

| Items | Remarks |
| --- | --- |
| Diameter | 1 mm |
| Length | 500 mm |
| Materials | High-temperature material |
| Temperature measurement range | 0-1100 °C |
| Measurement error | 0.4% |
| Layout method | Polyimide tape bonding |

**Table S7** Grid independence analysis

| Mesh scheme | Number of cells | *T*_max_ (°C) | relative error |
| --- | --- | --- | --- |
| Grid 1 | 232381 | 92.2 | - |
| Grid 2 | 327838 | 90.7 | 1.62% |
| Grid 3 | 490059 | 88.2 | 2.75% |
| Grid 4 | 961383 | 88.1 | ＜0.5% |

**Supplementary References**

[S1] W. Qiu, G. Li, C. Ouyang, J. Zeng, Simulation and experimental investigation of battery thermal management system for a hybrid vehicle. in: IOP Conf. Ser.: Earth Environ. Sci. (2021). <https://doi.org/10.1088/1755-1315/631/1/012093>
